# Comparative analysis of ligand binding affinities and functional profiles at recombinant human *β*-adrenergic receptor subtypes 1, 2, and 3

**DOI:** 10.1016/j.jpet.2026.104960

**Published:** 2026-06-08

**Authors:** Lilly Josephine Bindel, Roland Seifert

**Affiliations:** Institute of Pharmacology, Hannover Medical School, Hannover, Germany

**Keywords:** G protein-coupled receptor, *β*-Adrenergic receptor, IUPHAR, Binding affinity, Functional selectivit, Stereoisomer

## Abstract

*β*-Adrenergic receptors (*β*_x_AR) are G protein-coupled receptors with major physiological and therapeutic relevance in cardiovascular and respiratory conditions. Although numerous ligands have been investigated, binding affinities and functional annotations remain incomplete and fragmented across databases. This review systematically integrates ligand binding affinities and functional profiles at recombinant human *β*_x_AR from the Psychoactive Drug Screening Program K_i_ database and the International Union of Basic and Clinical Pharmacology/British Pharmacological Society (IUPHAR/BPS), with a focus on clinically important ligands, receptor selectivity, and characteristic patterns. Database agreement, affinity patterns, stereoisomer-specific information and concordance with primary literature are evaluated. In total, 156 ligands with pK_i_ ≥ 5 were collected. Median pK_i_ values were 6.6 for h*β*_1_AR, 6.15 for h*β*_2_AR, and 5.8 for h*β*_3_AR. A pronounced overlap between h*β*_1_AR and h*β*_2_AR was observed, reflected in similar median affinities and shared ligand profiles, whereas h*β*_3_AR showed narrower data coverage and a more distinct affinity distribution. Most ligands were listed for more than 1 h*β*_x_AR, and 37 ligands had binding data for all 3 h*β*_x_AR. Functional characterization was available for 90 ligands, whereas 61 lacked annotations. Database comparison revealed low overlap in ligand listings (28.8%). Stereoisomer data were highly incomplete, and in several cases, affinity-based stereoisomer comparisons differed from literature-reported stereoisomeric superiority. Summarized, substantial similarity between h*β*_1_AR and h*β*_2_AR ligand binding patterns is observed, whereas h*β*_3_AR appears less extensively characterized. Considerable fragmentation, incomplete receptor profiling and methodological heterogeneity limit the evaluation of receptor and ligand characteristics. Binding affinity and functional behavior must be interpreted as context-dependent parameters influenced by experimental systems, G protein coupling conditions and stereochemical specification.

**Significance Statement:**

This analysis systematically integrates ligand binding affinities and functional labels at recombinant human *β*_x_AR from the Psychoactive Drug Screening Program (PDSP) K_i_ database and the International Union of Basic and Clinical Pharmacology/British Pharmacological Society (IUPHAR/BPS) Guide to Pharmacology, providing a structured and comprehensive overview of ligand profiles. It identifies substantial data fragmentation, incomplete receptor and stereoisomer characterization, and limited concordance between databases. For clinically relevant ligands, it was demonstrated that therapeutic subtype classification does not always correspond to strict affinity-based receptor selectivity.

## 1 Introduction

*β*-Adrenergic receptors (*β*_x_AR) belong to the G protein-coupled receptor (GPCR) family, being expressed in numerous human organs and tissues where they modulate important biological effects,^1^ with adrenaline and noradrenaline being the endogenous ligands.^2^ Three human *β*_x_AR subtypes are known, which share a high degree of sequence and structural homology, particularly within the transmembrane domains that form the orthosteric binding pocket.^3^ This structural similarity provides a molecular basis for overlapping ligand binding and contributes to the frequently observed crossreactivity of a ligand between receptor subtypes.^4^ However, subtle differences in amino acid composition and receptor conformation can still result in subtype preference and functional divergence.^5^

Over the last decades, extensive research has led to the identification of many ligands acting as agonists, antagonists, and inverse agonists on h*β*_x_AR. Besides experimental investigation and comparison of compounds,^6^, ^7^, ^8^, ^9^ studies have analyzed the functional behavior of h*β*_x_AR and their ligands under varying biochemical and experimental conditions.^10^, ^11^, ^12^, ^13^, ^14^, ^15^ However, despite substantial experimental work and the investigation of clinically relevant ligands,^2^^,^^3^ the characterization of h*β*_x_AR ligands remains fragmented and incomplete. Binding affinity data and functional annotations are dispersed across different databases and primary literature sources, often without systematic crosscomparison.

**Fig. 1:**
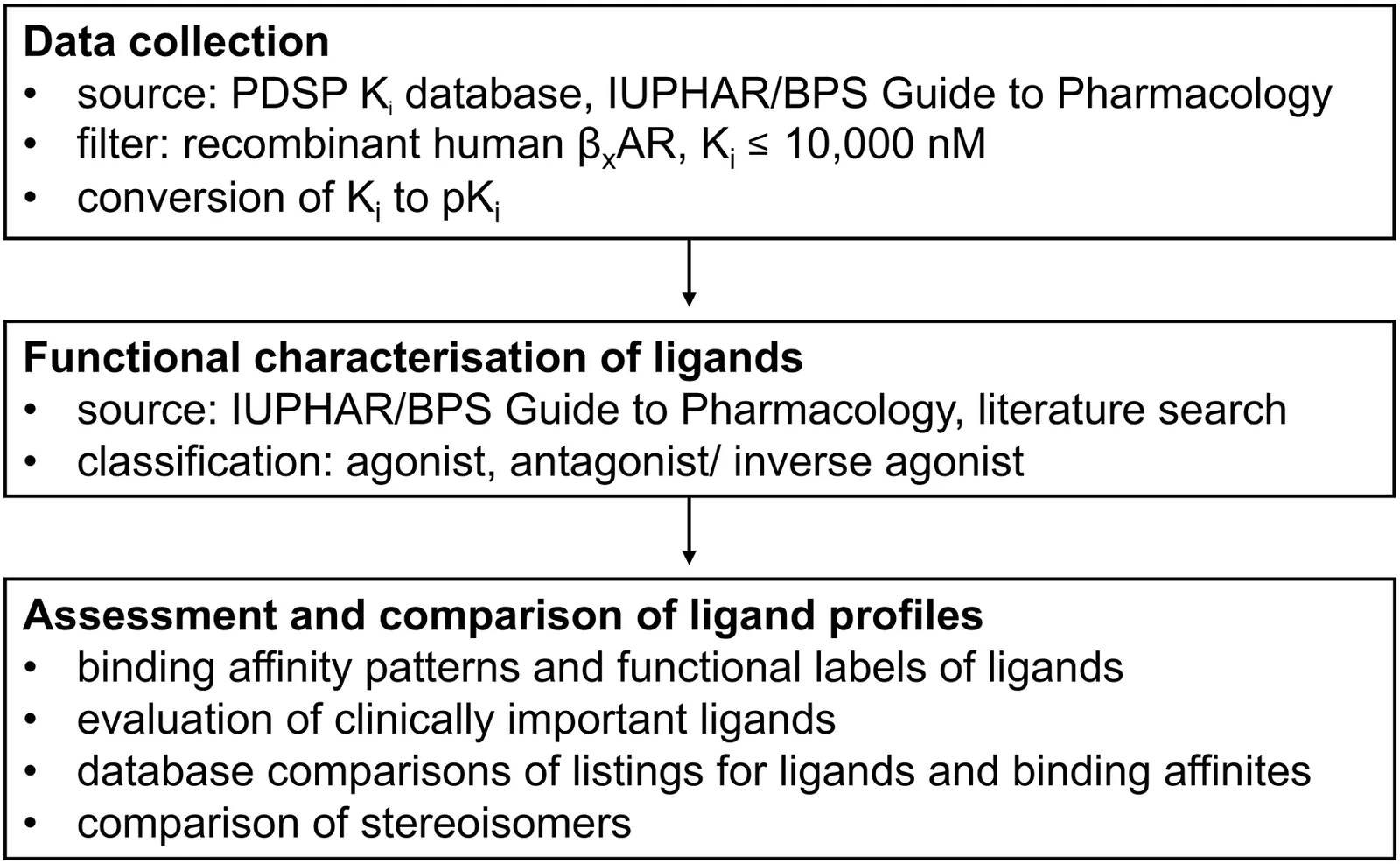
Flowchart of the methodological approach.

Therefore, binding affinity data and functional annotations for ligands targeting recombinant h*β*_x_AR were extracted from the Psychoactive Drug Screening Program (PDSP) K_i_ database^16^ and the International Union of Basic and Clinical Pharmacology/British Pharmacological Society (IUPHAR/BPS) Guide to Pharmacology^17^ (accessed February 2026), complemented by literature search. Stereoisomers were analyzed separately. In Fig. 1A flowchart visualizes methodological considerations.

This analysis aimed to systematically integrate and compare binding affinity data and functional characterization for recombinant h*β*_x_AR ligands. Specifically, it addresses the distribution of ligand binding affinities across h*β*_x_AR, subtype selectivity versus broader activity, the completeness and concordance of database entries, and the impact of stereochemistry and experimental context on apparent affinity and functional classification.^1^, ^2^, ^3^, ^4^, ^5^, ^6^, ^7^, ^8^, ^9^, ^10^, ^11^, ^12^, ^13^, ^14^, ^15^, ^16^, ^17^, ^18^, ^19^, ^20^, ^21^, ^22^, ^23^, ^24^, ^25^, ^26^, ^27^, ^28^, ^29^, ^30^, ^31^, ^32^, ^33^, ^34^, ^35^, ^36^, ^37^, ^38^, ^39^, ^40^, ^41^, ^42^, ^43^, ^44^, ^45^, ^46^, ^47^, ^48^, ^49^, ^50^, ^51^, ^52^, ^53^, ^54^, ^55^, ^56^, ^57^, ^58^, ^59^, ^60^, ^61^, ^62^, ^63^, ^64^, ^65^, ^66^, ^67^, ^68^, ^69^, ^70^, ^71^, ^72^, ^73^, ^74^, ^75^, ^76^ A structured overview of ligand binding patterns at recombinant h*β*_x_AR is provided, and methodological limitations influencing data interpretation are identified.

## 2 Background and therapeutic importance

Ligands acting at h*β*_x_AR are of high therapeutic relevance, particularly h*β*_1_AR and h*β*_2_AR antagonists, both as primary therapeutic drugs but also as contributors to adverse drug effects.^1^^,^^18^^,^^19^ The h*β*_1_AR primarily exerts cardiovascular effects, the h*β*_2_AR plays an important role on the respiratory system, and the h*β*_3_AR is being involved in gastrointestinal and metabolic regulation.^20^^,^^21^

The h*β*_1_AR is a central target in cardiovascular therapy.^22^ Agonists such as adrenaline (**2**) and noradrenaline (**122**) are used in acute settings including shock and cardiac arrest, whereas isoprenaline (**87**) is applied in bradycardia as short-term treatment. Antagonists reduce cardiac sympathetic activity and are standard therapy in heart failure, hypertension, and arrhythmia. Clinically important drugs include metoprolol (**102**), bisoprolol (**43**), carvedilol (**55**), nebivolol (**119**), labetalol (**95**), sotalol (**145**), and esmolol (**71**).^2^^,^^3^^,^^23^

The h*β*_2_AR is primarily targeted in respiratory diseases.^24^^,^^25^ Agonists with broncho-dilatory activity are indicated for asthma and chronic obstructive pulmonary disease. Short-acting drugs such as salbutamol (**140**) and terbutaline (**148**) provide rapid symptom relief, whereas long-acting drugs including formoterol (**80**) and salmeterol (**141**) are used for maintenance therapy. The clinical importance of subtype selectivity becomes evident from broncho-constrictive adverse effects observed with nonselective h*β*_x_AR antagonists,^2^^,^^3^ and therapeutically relevant differences in pharmacological properties of h*β*_2_AR agonists.^26^

The h*β*_3_AR has gained therapeutic relevance more recently. Agonists such as mirabegron (**103**) are approved for the treatment of overactive bladder, whereas additional substances such as solabegron (**144**) are under investigation.^3^^,^^20^^,^^21^ Table 1 provides an overview of exemplary h*β*_x_AR ligands with high clinical relevance.

**Table 1:** Selection of clinically important ligands at h*β*_x_AR

Drug	Ligand No.	Clinical Use^3^
Adrenaline (epinephrine)	**2**	Shock, anaphylaxis, cardiac arrest
Bisoprolol	**43**	Heart failure, hypertension
Carvedilol	**55**	Heart failure
Esmolol	**71**	Arrhythmia (iv, intensive care)
Isoprenaline	**87**	Bradycardia (short-term, bridge to pacemaker)
Labetalol	**95**	Hypertension (pregnancy), eclampsia
Metoprolol	**102**	Heart failure
Noradrenaline	**122**	Shock
Sotalol	**145**	Arrhythmia
Propranolol	**134**	Angina pectoris, heart failure, anxiety, migraine, benign essential tremor, thyrotoxicosis, portal hypertension, variceal bleeding
Mirabegron	**103**	Overactive bladder syndrome
Solabegron	**144**	Overactive bladder syndrome
Formoterol	**80**	Asthma/chronic obstructive pulmonary disease (COPD) (long-acting maintenance treatment)
Salbutamol	**140**	Asthma/COPD (short-acting rescue)
Salmeterol	**141**	Asthma/COPD (long-acting maintenance treatment)
Terbutaline	**148**	Asthma/COPD (short-acting rescue)

Provided is the drug name, the assigned ligand no. (highlighted in bold), and its established indication. Ligands are sorted by therapeutic category and ligand no. A visualization of the binding affinities of these ligands can be found in Fig. 5, whereas more information about binding affinities and physiological effects can be found in Tables 2 and 6. COPD, chronic obstructive pulmonary disease.

Beyond their therapeutic importance, *β*_x_AR have contributed substantially to general GPCR pharmacology. Studies on recombinant human receptors demonstrated constitutive receptor activity, inverse agonism, partial agonism, and system-dependent ligand behavior.^2^^,^^3^^,^^7^^,^^9^ More recent structural and molecular investigations further refined the understanding of receptor activation and functional selectivity.^10^^,^^15^

Although *β*_x_AR represent one of the most extensively studied GPCR families, ligand profiling across subtypes remains uneven. Many compounds are incompletely characterized across all 3 h*β*_x_AR, and stereoisomer-specific data are frequently lacking. These aspects underline the relevance of a systematic and comparative integration of available binding and functional data.

## 3 Binding affinities of h*β*_1_AR-h*β*_3_AR for ligands retrieved from the PDSP K_i_ database and the IUPHAR/BPS Guide to Pharmacology

For the human *β* adrenoreceptor family (h*β*_1_AR, h*β*_2_AR, and h*β*_3_AR), 156 ligands were extracted from the PDSP K_i_ database and the IUPHAR/BPS Guide to Pharmacology with binding affinities above pK_i_ ≥ 5 (K_i_ ≤ 10,000 nM).^16^^,^^17^ The included ligands comprise approved drugs, endogenous ligands, as well as experimental compounds. In Table 2, the binding affinities for all ligands and h*β*_x_AR are presented.

**Table 2:** Overview about binding affinities (pK_i_) and ligands at h*β*_x_AR, retrieved from the PDSP K_i_ database and the IUPHAR/BPS Guide to Pharmacology^16^^,^^17^

Ligand Name	Ligand No.	h*β*_1_AR pK_i_	h*β*_2_AR pK_i_	h*β*_3_AR pK_i_	h*β*_1_AR pK_i_ Range (Deviation Databases)	h*β*_2_AR pK_i_ Range (Deviation Databases)	h*β*_3_AR pK_i_ Range (Deviation Databases)
(−)-Ro 363	**1**	8.0					
(±)-Adrenaline (epinephrine)	**2**	6.0		Inactive			
(Â±)-(4aS 8R 8aS)-8-amino-2-cyclohexyl-3 4 4a 7 8 8a-hexahydroisoquinolin-1(2H)-one	**3**			5.2			
(Â±)-(4aS 8S 8aS)-2-benzyl-N-(4-chloro-3-(trifluoromethyl)phenyl)-1-oxo-1 2 3 4 4a 7 8 8a-octahydroisoquinoline-8-carboxamide	**4**			5.3			
(Â±)-2 5-Dimethoxy-4-bromoamphetamine	**5**	5.6	6.5				
(Â±)-2 5-Dimethoxy-4-ethylamphetamine	**6**	5.2	5.7				
(Â±)-2 5-Dimethoxy-4-iodoamphetamine	**7**	6.2	6.9				
(Â±)-2 5-Dimethoxy-4-methylamphetamine	**8**		7.3				
(Â±)-3 5-Dichloro-N-((4aS 8R 8aS)-2-cyclohexyl-1-oxo-1 2 3 4 4a 7 8 8a-octahydroisoquinolin-8-yl)benzamide	**9**			5.4			
(Â±)-4-Bromo-3-(trifluoromethyl)-N-((4aS 8R 8aS)-2-cyclohexyl-1-oxo-1 2 3 4 4a 7 8 8a-octahydroisoquinolin-8-yl)benzamide	**10**			5.0			
(Â±)-4-Bromo-3-(trifluoromethyl)-N-((4aS 8R 8aS)-2-cyclohexyl-1-oxo-1 2 3 4 4a 7 8 8a-octahydroisoquinolin-8-yl)benzamide	**11**			5.0			
(Â±)-4-Chloro-3-(trifluoromethyl)-N-((4aS 8R 8aS)-2-cyclohexyl-1-oxo-1 2 3 4 4a 7 8 8a-octahydroisoquinolin-8-yl)benzamide	**12**			5.2			
(Â±)-4-Chloro-N-(((4aS 8R 8aS)-2-benzyl-1 2 3 4 4a 7 8 8a-octahydroisoquinolin-8-yl)methyl)-3-(trifluoromethyl)aniline	**13**	5.7		6.0			
(Â±)-4-Ethylthio-2 5-dimethoxyamphetamine	**14**	5.2	7.6				
(Â±)-N-(((4aS 8R 8aS)-2-(3 4-dichlorobenzyl)-1 2 3 4 4a 7 8 8a-octahydroisoquinolin-8-yl)methyl)aniline	**15**			5.1			
(Â±)-N-((4aS 8R 8aS)-2-benzyl-1-oxo-1 2 3 4 4a 7 8 8a-octahydroisoquinolin-8-yl)-4-chloro-3-(trifluoromethyl)benzamide	**16**			5.2			
[^125^I]ICYP	**17**						
[^3^H]dihydroalprenolol	**18**						
[^3^H]L748337	**19**						
1-[4-(5-Chloro-2-methylphenyl)-1-piperazinyl]-3-(2 5-dimethylphenoxy)-2-propanol dihydrochloride	**20**	6.0	6.1	6.0			
1-1 (1-Fluoropropan-2-ylamino)-3-(20cyclohexlpheonxy)propan-2-ol ((2R 2S)-1)	**21**	8.2	8.7			0.0	
1-1 (1-Fluoropropan-2-ylamino)-3-(20cyclohexlpheonxy)propan-2-ol ((2S)-1)	**22**	8.2					
1-Methyl-7 8 9 10-tetrahydro-1H-6 10-methanoazepino[4 5-g] quinoxalin-2 (6H)-one HCl	**23**	5.3					
2-(Dimethylamino)-2-oxoethyl 4â€^2^-(Piperidin-4-yl)-5-(4-(4-(trifluoromethyl)phenyl)-1H-1 2 3-triazol-1-yl)-[1 1â€^2^-biphenyl]-3-carboxylate	**24**		5.4	5.9			
25CN-NBOH (N-(2-hydroxybenzyl)-4-cyano-2,5-dimethoxyphenethylamine)	**25**		5.8				
2-Cl-diphenidine	**26**	5.0	5.0	5.0			
2-Methoxy-diphenidine	**27**	5.0	5.0	5.0	0.0		
3-(Piperazin-1-yl)-N-(m-tolyl)propanamide	**28**	5.0	5.0				
3-Methoxy-diphenidine	**29**	5.0	5.0	5.0			
4-Ethylthio-2 5-dimethoxyphenethylamine	**30**		5.9				
4-Ethylthio-2 5-dimethoxyphenylbutylamine	**31**		6.9				
4-Methoxy-diphenidine	**32**	5.0	5.0	5.0			
6-APB (6-(2-aminopropyl)benzofuran)	**33**	5.4					
7-Methylcyanopindolol	**34**						
9-OH-risperidone	**35**	5.3	5.3				
Abediterol	**36**						
Acebutolol	**37**	6.5			0.1		
Alprenolol (±)	**38**	8.2	*9.0*	7.5		(0.1)	
Amibegron	**39**			5.2			
Atenolol	**40**	*6.8*	*5.4*	Inactive	0.8 (0.7)	0.4 (0.7)	
Betaxolol	**41**	8.7	7.7		0.9	1	
BI-167107	**42**						
Bisoprolol	**43**	*7.7*	5.9	5.0	(0.2)		
Brexpiprazole	**44**	6.2		5.8			
BRL 37344	**45**	*5.2*	*5.8*	*6.5*	(?)	(1.5)	0.6 (0.3)
BRL35135	**46**		7.4				
Bromocriptine	**47**	6.2	6.1				
Broxaterol	**48**	5.9	5.9	5.4			
Bucindolol	**49**	9.3					
Bunolol	**50**	8.4	9.3	6.8			
Bupranolol	**51**	8.2	9.1	7.1	0.7	1.6	
Butoxamine	**52**						
Carazolol	**53**		10.2	8.4		0.6	
Cariprazine	**54**	6.5		5.7			
Carvedilol	**55**	*9.0*	*9.3*	*7.7*	0.7 (0.4)	0.5 (0.7)	0.1 (2.2)
CGP 12177	**56**	*8.9*	*8.8*	*6.9*	(1.2)	(1.3)	(0.4)
CGP 20712-A	**57**	*8.2*	5.4	*5.5*	(0.3)		(0.2)
CHF-6366	**58**						
Cicloprolol	**59**	8.0	6.2				
Cimaterol	**60**	6.6		5.3			
Cis-2a	**61**	6.4	5.3				
CL316243	**62**			5.2			
Clenbuterol	**63**	7.7	7.7		0.5	0.5	
Clozapine	**64**	5.3	5.8				
Cyanopindolol	**65**						
D-578	**66**		Inactive	5.6			
Denopamine	**67**	6.0			0.3		
Diphenidine	**68**	5.0	5.0	5.0			
DKR-1677	**69**	5.2	Inactive	Inactive			
Epinephrine ((−)-adrenaline)	**70**	*5.5*	*5.9*	*Inactive*	(0.2)	(0.4)	(?)
Esmolol	**71**	6.8			0.2		
FEMPT	**72**	5.9	6.7	6.2			
Fenfluramine (±)	**73**	6.0	5.1				
Fenfluramine (−)	**74**	6.0	5.1				
Fenoterol (±)	**75**	5.0	*5.9*	*5.4*		(0.4)	(?)
Finazine	**76**	5.9	Inactive	Inactive			
Fluorocarazolol, (R)	**77**	9.3	9.9				
Fluorocarazolol, (S)	**78**	9.9	10.1				
Fluspiriline	**79**	6.2	6.3				
Formoterol (±)	**80**	*6.2*	5.9	5.4	(0.2)		
H87/07	**81**	7.0					
Haloperidol	**82**	Inactive	5.3				
ICI 118, 551	**83**	7.0	*9.3*	*6.2*		(0.1)	(0.0)
ICI 89, 406	**84**	8.8	6.9				
ICI-89406	**85**	8.8					
Indacaterol (R)	**86**	*7.5*	*7.9*	8.3	(1.5)	(0.5)	
Isoprenaline	**87**	6.6	6.5	5.7	0.9	0.2	1.1
Isoproterenol (−)	**88**		5.8				
Isoproterenol (+) (isoproterenol-l)	**89**	6.6	6.3	5.8			
KUR-1246	**90**	5.8	7.6	Inactive			
L 755507	**91**						
L742791	**92**						
L-748328	**93**			8.5			0.2
L-748337	**94**			8.2			0.4
Labetalol	**95**	7.9	8.0		0.6		
Landiolol	**96**	7.0					
Levosalbutamol	**97**	Inactive					
Lisuride	**98**	7.4	7.7				
LK 204-545	**99**	8.4	5.2		0.3		
Lysergic acid (+)	**100**	5.8	5.5				
mCPP	**101**	5.6	5.5				
Metoprolol (±)	**102**	*7.3*	*6.2*	Inactive	(0.2)	(0.8)	
Mirabegron	**103**						
MRP3160	**104**			8.2			
MRP3172	**105**	8.3	8.3				
MRP3215	**106**	8.2	8.2				
MRS5676	**107**		Inactive	5.7			
MRS5678	**108**		Inactive	5.8			
MRS5697	**109**		Inactive	5.9			
MRS5698	**110**		Inactive	5.8			
MRS5755	**111**		Inactive	5.6			
N-(3-chlorophenyl)-3-[4-(3-phenyl-2-propen-1-yl)-1-piperazinyl]propanamide	**112**	5.5					
N-(3-methylphenyl)-3-[4-(3-phenyl-2-propen-1-yl)-1-piperazinyl]propanamide hydrochloride	**113**	5.9					
Nadolol	**114**	6.9	7.8	6.3		1.6	0.1
Naftopidil	**115**	6.7	6.6	6.6			
NDD-713	**116**	8.2			0.7		
NDD-825	**117**	8.7			0.7		
N-desmethylclozapine	**118**	5.2	5.3				
Nebivolol	**119**	9.1	8.0	6.4		0.1	1.3
NIHP (1-[4-(3-hydroxypropyl)phenoxy]-3-(propan-2-ylamino)propan-2-ol)	**120**	7.1	6.0				
NIP (N-isopropyl-phenoxypropanolamine)	**121**	8.4	7.5				
Noradrenaline- (±) (norepinephrine- (±))	**122**	6.0	5.4				
Noradrenaline- (−) (norepinephrine- (−))	**123**	*5.6*	Inactive	*6.1*	(0.3)	0.8	(1.1)
Olanzapine	**124**	5.3	5.3				
Olodaterol	**125**						
Orciprenaline	**126**						
ORG-5222 (±) (asenapine (±))	**127**	5.3	5.3				
Pindolol (±)	**128**	*8.9*	*8.9*	*7.2*	(0.5)	(1.1)	(0.3)
Pipamerone	**129**	5.3	5.3				
Practolol	**130**	9.1					
Prenalterol	**131**	6.6					
Procaterol	**132**		7.1				
Propafenone	**133**	6.7	7.4				
Propranolol (±)	**134**	8.9	*9.4*	6.8		(0.1)	0.9
Propranolol l-	**135**	*8.6*	9.1	6.7	(0.3)		
Quetiapine	**136**	5.3	5.3				
Ractopamine	**137**		6.8				
Risperidone	**138**	5.3	5.3				
S S-2c	**139**	7.1	6.0				
Salbutamol (±) (albuterol)	**140**	5.9	*5.8*	8.3		(0.2)	
Salmeterol	**141**	6.1	*6.9*	5.4		(1.7)	
SB204070	**142**	6.0	6.0				
Sertindole	**143**	5.3	5.3				
Solabegron	**144**						
Sotalol	**145**	6.0	6.7		0.3		
SR 59230A	**146**	*7.9*	*8.1*	*7.3*	(0.3)	(1.7)	(0.7)
T-0509	**147**	8.1			1.1		
Terbutaline	**148**	*Inactive*	*5.6*	Inactive	(?)	(?)	
Terguride	**149**	6.2	7.7				
Tertatolol	**150**			8.6			
Timolol	**151**	8.7	9.7				
TMQ (±)-8-fluoro (8-fluorotrimetoquinol)	**152**	7.4	8.1				
Vibegron	**153**						
Vilanterol	**154**						
Xamoterol	**155**	7.1			0.2		
Zinterol	**156**		8.0				

If duplicate values within one database are available, the median is given and the range is provided. If binding affinities for one ligand at one h*β*_x_AR were available in both databases, the merged binding affinity (median) is given in italics, along with the deviation between both median values for the range in parentheses. The label “inactive” is assigned to ligands where available data report a binding affinity above K_i_ 10,000 nM, whereas the label “no data” highlights that no binding affinity is available in any database. Ligands are sorted in ascending order of their ligand no. (highlighted in bold).

Most K_i_ values included in this analysis are derived from radioligand binding assays, based on competitive displacement of radioactively labeled ligands by the test compound. Frequently used radioligands in the compiled h*β*_x_AR dataset include [^125^I]CYP ([^125^I]cyanopindolol) and [^3^H]CGP 12177 for all h*β*_x_AR, and [^3^H]DHA ([^3^H]dihydroalprenolol) for h*β*_2_AR. [^125^I]CYP was used in binding experiments including ligands such as propranolol (**134**) and atenolol (**40**), [^3^H]CGP12177 was used in studies including lisuride (**98**) and terguride (**149**), and [^3^H]DHA was used in mCPP (**101**) and olanzapine (**124**). In Supplemental Table 1, frequently used radioligands are characterized for h*β*_x_AR by pK_d_ values. Because radioligand affinity directly influences assay suitability, signal-to-noise ratio and the precision of derived pK_i_ values, radioligand identity represents an important methodological aspect in binding affinity studies.

Most ligands (*n* = 64, 41.0%) are only listed for one h*β*_x_AR subtype. However, multiple ligands have available binding affinities for several h*β*_x_AR, for 2 receptors (*n* = 55, 35.3%), and for all 3 receptors (*n* = 37, 23.7%). Regarding the availability of binding affinities in ligands, the highest share is observed for the h*β*_1_AR (*n* = 115, 73.7%), followed by h*β*_2_AR (*n* = 105, 67.3%), and h*β*_3_AR (*n* = 47, 30.1%) (Supplemental Table 2).

Regarding the distribution of the binding affinities, the highest average (median) pK_i_ is observed for the h*β*_1_AR (6.6), followed by the h*β*_2_AR (6.15), and h*β*_3_AR (5.8). A wide range of binding affinity values is observed for the h*β*_2_AR and h*β*_1_AR, with 5.2 and 4.9 pK_i_ (min–max: 5.0–10.2; 5.0–9.9), respectively. A comparable narrow range, although with a lower maximum binding affinity, is observed for the h*β*_3_AR (pK_i_ range 3.6; 5.0–8.6) (Supplemental Table 2). The similarities in median pK_i_ values across h*β*_1_AR and h*β*_2_AR suggest a partially overlapping affinity, which is consistent with their high sequence homology and conserved structural features within the ligand binding pocket.^27^ However, the broader range observed may reflect pharmacological diversity of ligands or heterogeneous experimental conditions.

**Fig. 2:**
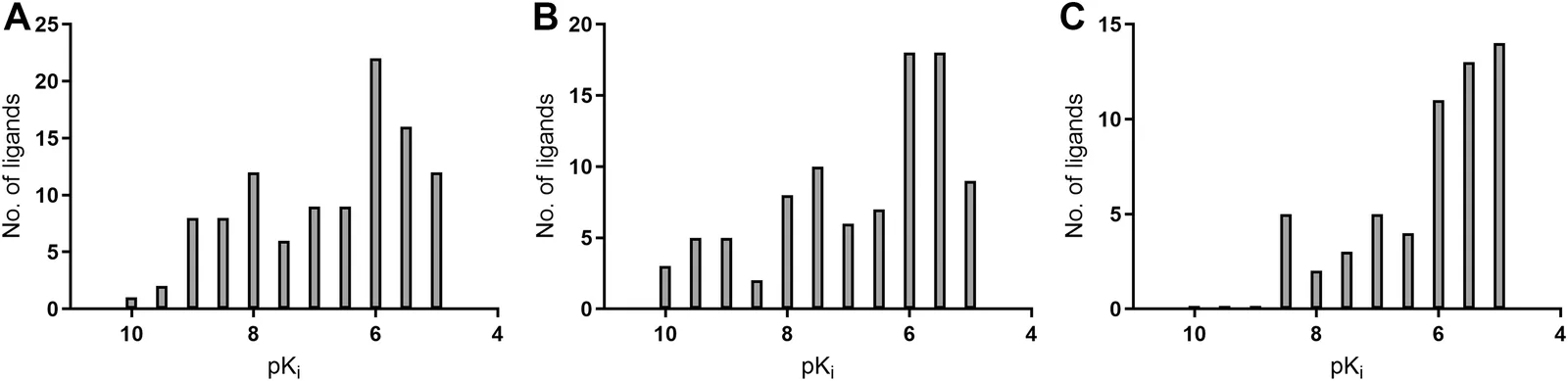
Distribution of binding affinities (pK_i_) of ligands at h*β*_x_AR. Separate panels are shown for (A) h*β*_1_AR, (B) h*β*_2_AR, and (C) h*β*_3_AR. Gray-colored bars represent the absolute number of ligands per binding affinity value (pK_i_).

The distribution of ligand binding affinities across the 3 h*β*_x_AR is summarized in Fig. 2 and Supplemental Fig. 1. To account for substantial differences in data availability between h*β*_x_AR subtypes, binding affinities are shown both as absolute ligand counts and as relative percentage distributions. The broader distributions for h*β*_1_AR and h*β*_2_AR and the more confined distributions for h*β*_3_AR reflect the differences in pK_i_ ranges and median values.

## 4 Characterization of ligands as agonists, antagonists, and inverse agonists at recombinant h*β*_x_AR

For each ligand, a functional label was assigned if available. This includes a characterization of receptor-specific agonist, antagonist, and inverse agonist behavior on h*β*_x_AR. Therefore, databases and corresponding literature were searched. Preferably, the annotation provided by the IUPHAR/BPS Guide to Pharmacology was referred to. If no functional label had been assigned to a specific ligand, the primary literature linked in the PDSP K_i_ database was analyzed. If binding behavior was not characterized there either, an additional literature search in PubMed and PubChem was conducted.

For 90 ligands and 179 pK_i_ values, it was possible to obtain a functional characterization from literature. However, for 61 ligands and 113 pK_i_ values, no information was found. Notably, functional characterization of active binding on h*β*_x_AR was either completely available or missing for all corresponding pK_i_ values.

Table 3 presents the functional characterization of the selected ligands and h*β*_x_AR. The distinction between antagonistic and inverse agonistic behavior, and even between agonistic and antagonistic behavior, is not always consistent. Notably, some ligands are characterized both as agonists and antagonists at specific h*β*_x_AR, for example, CGP 12177 (**56**) and practolol (**130**) for h*β*_1_AR, Pindolol (±) (**128**) for h*β*_1_AR and h*β*_3_AR, as well as alprenolol (±) (**38**) for h*β*_2_AR. Many ligands historically assigned as agonists may act as inverse agonists under experimental conditions with measurable constitutive receptor activity, as observed for pindolol (**128**).^2^ Since the differentiation between antagonists and inverse agonists is assay-dependent and may not have been part of the original experimental design, this introduces some ambiguity into the functional classification. Importantly, this is not merely a limitation of reporting, but reflects fundamental principles of GPCR systems, where ligand behavior depends on receptor state and assay configuration rather than representing consistent intrinsic properties.^3^^,^^6^^,^^8^^,^^11^^,^^14^^,^^15^^,^^52^

**Table 3:** Functional characterization of ligands as agonists, antagonist, and inverse agonists at the h*β*_x_AR for “active” binding

Ligand Name	Ligand No.	h*β*_1_AR	h*β*_2_AR	h*β*_3_AR
(−)-Ro 363	**1**	Agonist^17^		
(±)-Adrenaline (epinephrine)	**2**	Agonist/ full agonist^17^		Agonist/full agonist^17^
(Â±)-(4aS 8R 8aS)-8-amino-2-cyclohexyl-3 4 4a 7 8 8a-hexahydroisoquinolin-1(2H)-one	**3**			?^28^
(Â±)-(4aS 8S 8aS)-2-benzyl-N-(4-chloro-3-(trifluoromethyl)phenyl)-1-oxo-1 2 3 4 4a 7 8 8a-octahydroisoquinoline-8-carboxamide	**4**			?^28^
(Â±)-2 5-Dimethoxy-4-bromoamphetamine	**5**	?^29^	?^29^	
(Â±)-2 5-Dimethoxy-4-ethylamphetamine	**6**	?^29^	?^29^	
(Â±)-2 5-Dimethoxy-4-iodoamphetamine	**7**	?^29^	?^29^	
(Â±)-2 5-Dimethoxy-4-methylamphetamine	**8**		?^29^	
(Â±)-3 5-Dichloro-N-((4aS 8R 8aS)-2-cyclohexyl-1-oxo-1 2 3 4 4a 7 8 8a-octahydroisoquinolin-8-yl)benzamide	**9**			?^28^
(Â±)-4-bromo-3-(trifluoromethyl)-N-((4aS 8R 8aS)-2-cyclohexyl-1-oxo-1 2 3 4 4a 7 8 8a-octahydroisoquinolin-8-yl)benzamide	**10**			?^28^
(Â±)-4-bromo-3-(trifluoromethyl)-N-((4aS 8R 8aS)-2-cyclohexyl-1-oxo-1 2 3 4 4a 7 8 8a-octahydroisoquinolin-8-yl)benzamide	**11**			?^28^
(Â±)-4-Chloro-3-(trifluoromethyl)-N-((4aS 8R 8aS)-2-cyclohexyl-1-oxo-1 2 3 4 4a 7 8 8a-octahydroisoquinolin-8-yl)benzamide	**12**			?^28^
(Â±)-4-Chloro-N-(((4aS 8R 8aS)-2-benzyl-1 2 3 4 4a 7 8 8a-octahydroisoquinolin-8-yl)methyl)-3-(trifluoromethyl)aniline	**13**	? (PDSP Certified Data)		? (PDSP Certified Data)
(Â±)-4-Ethylthio-2 5-dimethoxyamphetamine	**14**	?^29^	?^29^	
(Â±)-N-(((4aS 8R 8aS)-2-(3 4-dichlorobenzyl)-1 2 3 4 4a 7 8 8a-octahydroisoquinolin-8-yl)methyl)aniline	**15**			?^28^
(Â±)-N-((4aS 8R 8aS)-2-benzyl-1-oxo-1 2 3 4 4a 7 8 8a-octahydroisoquinolin-8-yl)-4-chloro-3-(trifluoromethyl)benzamide	**16**			?^28^
[^125^I]ICYP	**17**	Antagonist^17^	Antagonist^17^	Agonist/partial agonist^17^
[^3^H]dihydroalprenolol	**18**		Antagonist^17^	
[^3^H]L748337	**19**			Antagonist^17^
1-[4-(5-Chloro-2-methylphenyl)-1-piperazinyl]-3-(2 5-dimethylphenoxy)-2-propanol dihydrochloride	**20**	?^30^	?^30^	?^30^
1-1 (1-Fluoropropan-2-ylamino)-3-(20cyclohexlpheonxy)propan-2-ol ((2R 2S)-1)	**21**	?^31^	?^31^	
1-1 (1-Fluoropropan-2-ylamino)-3-(20cyclohexlpheonxy)propan-2-ol ((2S)-1)	**22**	?^31^		
1-Methyl-7 8 9 10-tetrahydro-1H-6 10-methanoazepino[4 5-g] quinoxalin-2 (6H)-one HCl	**23**	?^32^		
2-(Dimethylamino)-2-oxoethyl 4â€^2^-(Piperidin-4-yl)-5-(4-(4-(trifluoromethyl)phenyl)-1H-1 2 3-triazol-1-yl)-[1 1â€^2^-biphenyl]-3-carboxylate	**24**		?^33^	?^33^
25CN-NBOH (N-(2-hydroxybenzyl)-4-cyano-2,5-dimethoxyphenethylamine)	**25**		?^34^	
2-Cl-diphenidine	**26**	?^35^	?^35^	?^35^
2-Methoxy-diphenidine	**27**	?^35^	?^35^	?^35^
3-(Piperazin-1-yl)-N-(m-tolyl)propanamide	**28**	?^30^	?^30^	
3-Methoxy-diphenidine	**29**	?^35^	?^35^	?^35^
4-Ethylthio-2 5-dimethoxyphenethylamine	**30**		?^29^	
4-Ethylthio-2 5-dimethoxyphenylbutylamine	**31**		?^29^	
4-Methoxy-diphenidine	**32**	?^35^	?^35^	?^35^
6-APB ((6-(2-aminopropyl)benzofuran))	**33**	?^36^		
7-Methylcyanopindolol	**34**	Antagonist^17^	Antagonist/inverse agonist^17^	
9-OH-risperidone	**35**	?^37^	?^37^	
Abediterol	**36**	Agonist^17^	Agonist/full agonist^17^	Agonist^17^
Acebutolol	**37**	Antagonist^17^		
Alprenolol (±)	**38**	Antagonist^2^	Antagonist/partial agonist^17^	Agonist^2^
Amibegron	**39**			Agonist/full agonist^17^
Atenolol	**40**	Antagonist^17^	Antagonist^17^	Inactive (PDSP Certified Dat)
Betaxolol	**41**	Antagonist^17^	Antagonist^17^	
BI-167107	**42**	Agonist^17^	Agonist^17^	
Bisoprolol	**43**	Antagonist^17^	Antagonist^2^	Antagonist^2^
Brexpiprazole	**44**	? (PDSP Certified Data)		? (PDSP Certified Data)
BRL 37344	**45**	Agonist/partial agonist^17^	Agonist/partial agonist^17^	Agonist/partial-full agonist^17^
BRL35135	**46**		Agonist/partial agonist^17^	
Bromocriptine	**47**	?^38^	?^38^	
Broxaterol	**48**	Antagonist^2^	Agonist/partial agonist^2^	Agonist^2^
Bucindolol	**49**	Antagonist^17^		
Bunolol	**50**	Antagonist^17^	Antagonist^17^	Antagonist^17^
Bupranolol	**51**	Antagonist^17^	Antagonist^17^	Antagonist^17^
Butoxamine	**52**		Antagonist^17^	
Carazolol	**53**	Antagonist^17^	Antagonist^17^	Agonist/partial agonist^17^
Cariprazine	**54**	? (PDSP Certified Data)		? (PDSP Certified Data)
Carvedilol	**55**	Antagonist^17^	Antagonist^17^	Antagonist^17^
CGP 12177	**56**	Agonist/partial agonist; antagonist^17^	Antagonist^17^	Agonist/partial agonist^17^
CGP 20712-A	**57**	Antagonist^17^	Antagonist^2^	Antagonist^17^
CHF-6366	**58**		Agonist^17^	
Cicloprolol	**59**	Antagonist^17^	Antagonist^17^	
Cimaterol	**60**	Agonist^17^	Agonist^17^	Agonist/full agonist^17^
Cis-2a	**61**	?^29^	?^29^	
CL316243	**62**			Agonist^17^
Clenbuterol	**63**	Agonist/full agonist^17^	Agonist/full agonist^17^	
Clozapine	**64**	?^37^	?^37^	
Cyanopindolol	**65**	Antagonist^17^		
D-578	**66**		Inactive (PDSP)	?^39^
Denopamine	**67**	Agonist/partial agonist^17^		
Diphenidine	**68**	?^35^	?^35^	?^35^
DKR-1677	**69**	?^40^	Inactive (PDSP)	Inactive (PDSP)
Epinephrine ((−)-adrenaline)	**70**	Agonist^17^	Agonist/full agonist^17^	Agonist/full agonist^17^
Esmolol	**71**	Antagonist^17^		
FEMPT	**72**	?^41^	?^41^	?^41^
Fenfluramine (±)	**73**	? (PDSP Certified Data)	? (PDSP Certified Data)	
Fenfluramine (−)	**74**	? (PDSP Certified Data)	? (PDSP Certified Data)	
Fenoterol (±)	**75**	Agonist^17^	Agonist^17^	Agonist/full agonist^17^
Finazine	**76**	?^30^	Inactive (PDSP Certified Data)	Inactive (PDSP)
Fluorocarazolol, (R)	**77**	Antagonist^42^	Antagonist^42^	
Fluorocarazolol, (S)	**78**	Antagonist^42^	Antagonist^42^	
Fluspiriline	**79**	?^37^	?^37^	
Formoterol (±)	**80**	Agonist^17^	Agonist^17^	Agonist/full agonist^43^
H87/07	**81**	Antagonist^17^		
Haloperidol	**82**	Inactive (PDSP Certified Data)	?^37^	
ICI 118, 551	**83**	Antagonist^2^	Antagonist/inverse agonist^17^	Antagonist/inverse agonist^2^
ICI 89, 406	**84**	Agonist/partial agonist^17^	Agonist/partial agonist^44^	
ICI-89406	**85**	Agonist/partial agonist^17^		
Indacaterol (R)	**86**	Agonist^17^	Agonist^17^	Agonist/full agonist^43^
Isoprenaline	**87**	Agonist/full agonist^17^	Agonist/full agonist^17^	Agonist/full agonist^17^
Isoproterenol (−)	**88**		Agonist/partial agonist^44^	
Isoproterenol (+) (isoproterenol-l)	**89**	Antagonist^2^	Agonist/partial agonist^2^	Agonist^2^
KUR-1246	**90**	Agonist^45^	Agonist^45^	Inactive (PDSP Certified Data)
L 755507	**91**			Agonist/full agonist^17^
L742791	**92**			Agonist
L-748328	**93**			Antagonist^17^
L-748337	**94**			Antagonist^17^
Labetalol	**95**	Antagonist^17^	Agonist/partial agonist^17^	
Landiolol	**96**	Antagonist^17^		
Levosalbutamol	**97**	Agonist/partial agonist^17^		
Lisuride	**98**	?^38^	?^38^	
LK 204-545	**99**	Antagonist^17^	Antagonist^17^	
Lysergic acid (+)	**100**	?^29^	?^29^	
mCPP	**101**	? (PDSP Certified Data)	? (PDSP Certified Data)	
Metoprolol (±)	**102**	Antagonist^17^	Antagonist^17^	Inactive (PDSP Certified Data)
Mirabegron	**103**	Agonist^17^	Agonist^17^	Agonist^17^
MRP3160	**104**			?^46^
MRP3172	**105**	?^46^	?^46^	
MRP3215	**106**	?^46^	?^46^	
MRS5676	**107**		Inactive (PDSP Certified Data)	?^47^
MRS5678	**108**		Inactive (PDSP Certified Data)	?^47^
MRS5697	**109**		Inactive (PDSP Certified Data)	?^47^
MRS5698	**110**		Inactive (PDSP Certified Data)	?^47^
MRS5755	**111**		Inactive (PDSP Certified Data)	?^47^
N-(3-chlorophenyl)-3-[4-(3-phenyl-2-propen-1-yl)-1-piperazinyl]propanamide	**112**	?^30^		
N-(3-methylphenyl)-3-[4-(3-phenyl-2-propen-1-yl)-1-piperazinyl]propanamide hydrochloride	**113**	?^30^		
Nadolol	**114**	Antagonist^17^	Antagonist^17^	Antagonist^17^
Naftopidil	**115**	?^30^	?^30^	?^30^
NDD-713	**116**	Antagonist^17^		
NDD-825	**117**	Antagonist^17^		
N-desmethylclozapine	**118**	? (PDSP Certified Data)	? (PDSP Certified Data)	
Nebivolol	**119**	Antagonist^17^	Antagonist^17^	Antagonist^17^
NIHP (1-[4-(3-hydroxypropyl)phenoxy]-3-(propan-2-ylamino)propan-2-ol)	**120**	Antagonist^17^	Antagonist^17^	
NIP (N-isopropyl-phenoxypropanolamine)	**121**	Antagonist^17^	Antagonist^17^	
Noradrenaline- (±) (norepinephrine- (±))	**122**	Agonist^17^	Agonist/full agonist^17^	
Noradrenaline- (−) (norepinephrine- (−))	**123**	Agonist^17^	Agonist^17^	Agonist/full agonist^17^
Olanzapine	**124**	Antagonist^48^	Antagonist^48^	
Olodaterol	**125**		Agonist^17^	
Orciprenaline	**126**		Agonist^17^	
ORG-5222 (±) (asenapine (±))	**127**	?^37^	?^37^	
Pindolol (±)	**128**	Antagonist; agonist/partial agonist^17^	Antagonist^17^	Antagonist; partial agonist^17^
Pipamerone	**129**	?^37^	?^37^	
Practolol	**130**	Antagonist; partial agonist^17^		
Prenalterol	**131**	Agonist/partial agonist^17^		
Procaterol	**132**		Agonist^17^	
Propafenone	**133**	Antagonist^17^	Antagonist^17^	
Propranolol (±)	**134**	Antagonist^49^	Antagonist^17^	Antagonist^17^
Propranolol l-	**135**	Antagonist^17^	Antagonist^50^	Antagonist^2^
Quetiapine	**136**	?^37^	?^37^	
Ractopamine	**137**		Agonist/partial agonist^17^	
Risperidone	**138**	?^37^	?^37^	
S S-2c	**139**	?^29^	?^29^	
Salbutamol (±) (albuterol)	**140**	Antagonist^2^	Agonist/partial agonist^17^	Agonist^2^
Salmeterol	**141**	Antagonist/inverse agonist^2^	Agonist/partial agonist^17^	Antagonist/inverse agonist^2^
SB204070	**142**	?^51^	?^51^	
Sertindole	**143**	?^37^	?^37^	
Solabegron	**144**	Agonist^17^	Agonist^17^	Agonist^17^
Sotalol	**145**	Antagonist^17^	Antagonist^17^	
SR 59230A	**146**	Antagonist^17^	Antagonist^17^	Antagonist^17^
T-0509	**147**	Agonist/full agonist^17^		
Terbutaline	**148**	Agonist/partial agonist^17^	Agonist/partial agonist^17^	Inactive (PDSP Certified Data)
Terguride	**149**	?^51^	?^51^	
Tertatolol	**150**			Antagonist^17^
Timolol	**151**	Antagonist^17^	Antagonist^17^	
TMQ (±)-8-fluoro (8-fluorotrimetoquinol)	**152**	Agonist/partial agonist^44^	Agonist/partial agonist^44^	
Vibegron	**153**			Agonist^17^
Vilanterol	**154**		Agonist^17^	
Xamoterol	**155**	Agonist/partial agonist^17^		
Zinterol	**156**		Agonist^17^	

“?” stands for noncharacterized and therefore uncertain behavior in the literature. Agonists are green-colored, and antagonists and inverse agonists are orange-colored. If the ligand is characterized with both agonistic and antagonistic behavior, it is colored yellow. Ligands are sorted in ascending order of their ligand no. (highlighted in bold).

## 5 Ligand binding patterns across recombinant h*β*_x_AR

Ligands acting on h*β*_x_AR differ in both binding affinity (pK_i_) and functional behavior (agonist, antagonist, and inverse agonist). A distinction can be made between selective and nonselective ligands, as well as between uniform agonist/antagonist behavior and functional behavior across receptor subtypes. The patterns described here primarily emerge from the integration of PDSP K_i_ binding data with IUPHAR/BPS functional annotations (Table 3).

Selective ligands have a high affinity at one particular h*β*_x_AR but low affinity at others. A ligand was considered selective for one h*β*_x_AR subtype if the difference in binding affinity between subtypes was at least one logarithmic unit (ΔpK_i_ ≥ 1), corresponding to a ≥10-fold difference in K_i_ values. For the h*β*_1_AR, both selective agonists as well as antagonists/inverse agonists are observed. Ligands with an assigned antagonistic behavior and high selectivity for the h*β*_1_AR include bucindolol (**49**) and NDD-825 (**117**),^53^^,^^54^ whereas selective agonists are ICI-89406 (**85**), T-0509 (**147**), and Ro 363 (−) (**1**).^55^, ^56^, ^57^ Notably, there is a high number of h*β*_1_AR antagonists within retrieved ligands, attributable to their important therapeutical role in the treatment of cardiovascular conditions,^6^^,^^58^ which may have provided a strong incentive for experimental investigation and profiling. However, some ligands display a switching agonist/antagonist behavior, depending on the experimental setting, for example, practolol (**130**).^17^ Further ligands with high binding affinity are not characterized yet, such as 1-1(1-Fluoropropan-2-ylamino)-3-(20cyclohexlpheonxy)propan-2-ol ((2S)-1) (**22**).^31^

For the h*β*_2_AR, there is a lower number of selective ligands with lower binding affinity in comparison with h*β*_1_AR. Notably, the highest binding affinities for h*β*_2_AR are observed in ligands also displaying high pK_i_ values for h*β*_1_AR. Ligands that can be described as selective for the h*β*_2_AR include primarily agonists such as procaterol (**132**).^59^ However, some ligands with comparable high selective binding affinity for h*β*_2_AR are not characterized yet, such as (Â±)-2 5-Dimethoxy-4-methylamphetamine (**8**) and 4-ethylthio-2 5-dimethoxyphenylbutylamine (**31**).^29^ h*β*_2_AR agonists have a large therapeutical relevance in respiratory diseases because of their broncho-dilatory effect.^1^

For the h*β*_3_AR, selective ligands with high binding affinities include antagonists, for example, L-748328 (**93**) and L-748337 (**94**),^31^^,^^54^ and agonists, for example, amibegron (**39**),^19^ with some ligands such as MRP3160 (**104**) lacking functional labeling. h*β*_3_AR agonists are approved as therapeutic drugs for the treatment of overactive bladder, for example, mirabegron.^19^^,^^20^^,^^60^ h*β*_3_AR antagonists are not yet used in clinical practice, but investigated for their potential beneficial role in the treatment of cardiovascular diseases, obesity, and inflammatory conditions.^14^^,^^60^

Although it is likely that some ligands appear to be highly selective, this interpretation has to be made cautiously. In many cases, apparent selectivity may arise from incomplete receptor profiling rather than verified subtype specificity, since binding affinities are not available for all h*β*_x_AR in all ligands. Consequently, the absence of reported binding does not allow discrimination between true selectivity or incomplete experimental characterization. Therefore, the selectivity between h*β*_1_AR and h*β*_2_AR were exemplarily analyzed for ligands with available binding affinities for both receptor subtypes. Several ligands (*n* = 61) were nonselective for either h*β*_1_AR or h*β*_2_AR, with ΔpK_i_ < 1, for example, labetalol (**95**), clenbuterol (**63**), olanzapine (**124**), isoprenaline (**87**), pindolol (±) (**128**), or salbutamol (±) (**140**). In contrast, 5 ligands were selective (ΔpK_i_ > 1) for the h*β*_2_AR over h*β*_1_AR, for example, timolol (**151**) and ICI 118, 551 (**83**), whereas 11 ligands showed h*β*_1_AR preference, including atenolol (**40**) and bisoprolol (**43**). Supplemental Table 3 provides an overview about selective ligands between h*β*_1_AR and h*β*_2_AR.

The majority of ligands display multireceptor affinity. Frequent combinations include a high affinity on h*β*_1_AR and h*β*_2_AR, as well as on all 3 h*β*_x_AR. Notably, ligands having a high binding affinity on h*β*_1_AR and h*β*_2_AR are often acting as antagonists on both receptors, for example, fluorocarazol (S, R) (**77, 78**), and timolol (**151**).^61^ However, there are also dual agonists, for example, clenbuterol (**63**) and KUR-1246 (**90**).^45^^,^^62^ These combinations are of therapeutical relevance, since they explain and predict broncho-constricting adverse effects in the treatment of cardiovascular conditions with h*β*_1_AR antagonists, and cardiovascular adverse effects (tachycardia and myocardial infarction) in bronchopulmonary treatment with h*β*_2_AR agonists leading to increased mortality.^3^^,^^18^^,^^63^ Importantly, these patterns illustrate that multireceptor activity is not an exception but rather a common pharmacological feature of h*β*_x_AR ligands. Furthermore, switching behavior is sometimes observed, for example, labetalol (**95**), acting as an antagonist at the h*β*_1_AR and as an agonist at the h*β*_2_AR.^17^ A considerable number ligands with high binding affinities on the h*β*_1_AR and h*β*_2_AR are not characterized yet, for example, 1-1(1-fluoropropan-2-ylamino)-3-(20cyclohexlpheonxy)-propan-2-ol ((2R 2S)-1) (**21**), MRP3172 (**105**), and MRP3215 (**106**).^31^^,^^46^

Unselective behavior with active binding on all 3 h*β*_3_AR is another often observed pattern. Ligands with comparably high binding affinities are primarily antagonists at all 3 h*β*_x_AR, for example, propranolol (± and −) (**134, 135**) or carvedilol (**55**).^64^^,^^65^ Agonistic behavior at all 3 h*β*_x_AR is also observed, for example in isoprenaline (**87**), fenoterol (±) (**75**), and formoterol (±) (**80**).^2^ Functional selectivity is also observed, with ligands acting agonistic at 1 h*β*_x_AR and antagonistic at another h*β*_x_AR, for example, carazolol (**53**), salmeterol (**141**), or isoproterenol (+) (**89**).^17^ Again, several ligands with high binding affinities on all 3 h*β*_x_AR are not characterized yet, for example, FEMPT (**72**) or naftopidil (**115**).^16^^,^^30^

**Fig. 3:**
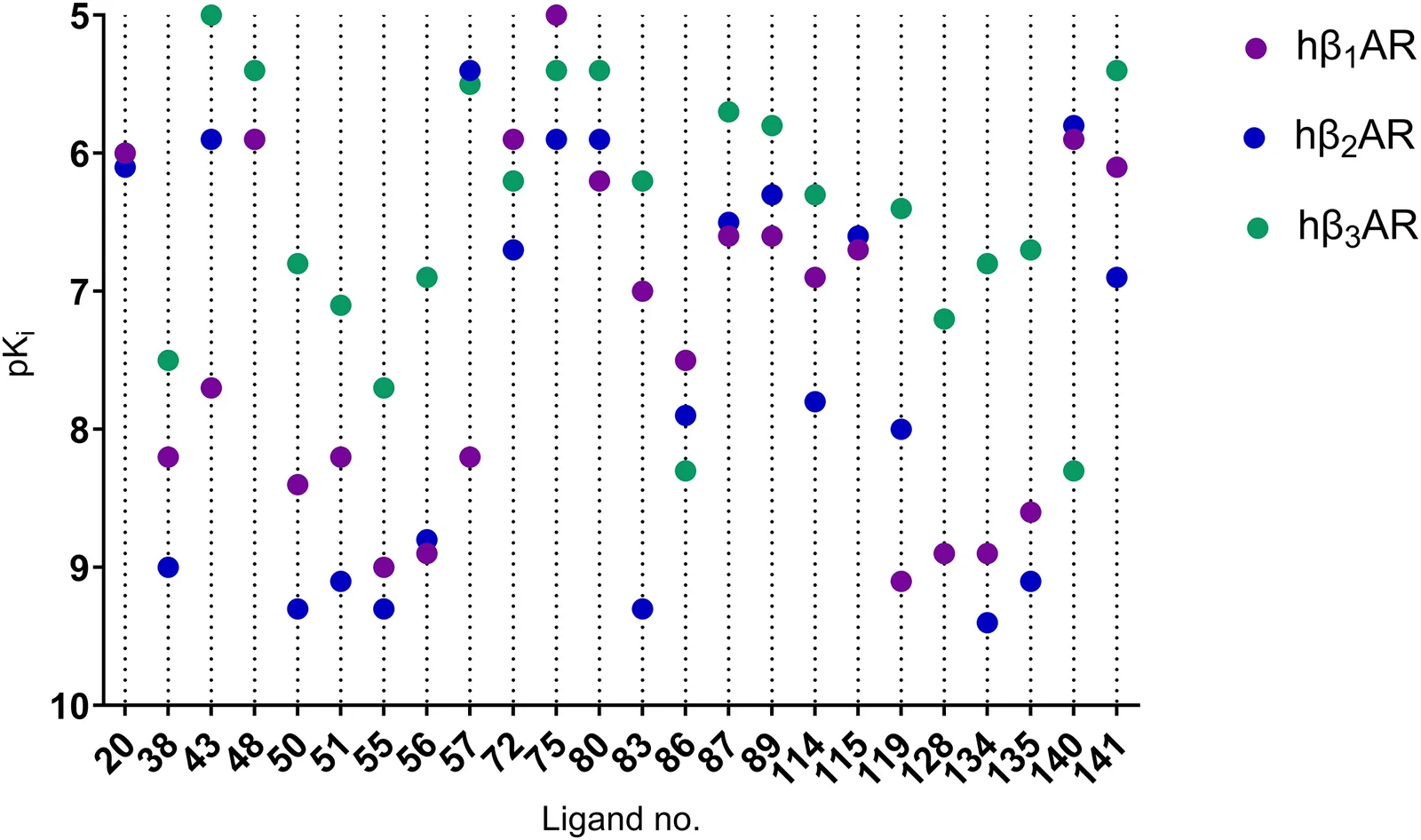
Broad-spectrum ligands with binding affinity data available for all 3 h*β*_x_AR subtypes. Each ligand is represented by its assigned ligand number (*x*-axis), and pK_i_ values are plotted on the *y*-axis. Ligands are sorted by its ligand no.

Figure 3 depicts ligands with broad binding affinity and visualizes their relative pK_i_ values across several h*β*_x_AR, whereas Table 4 summarizes all ligands with complete characterization. Notably, ligands are heterogeneous, ranging from relatively balanced multireceptor affinities, as seen for indacaterol (R) (**86**), formoterol (±) (**80**), FEMPT (**72**), or fenoterol (±) (**75**), to pronounced receptor-specific profiles, for example, in nevibolol (**119**) and bisoprolol (**43**).

**Table 4:** Overview about ligands with complete characterization at h*β*_x_AR, including binding affinities and functional labeling for all 3 h*β*_x_AR subtypes

Ligand Name		Ligand No.	Characterization			Binding Affinity (pK_i_)		
			h*β*_1_AR	h*β*_2_AR	h*β*_3_AR	h*β*_1_AR	h*β*_2_AR	h*β*_3_AR
Alprenolol (±)	**38**	Antagonist^2^	Antagonist/partial agonist^17^		Agonist^2^	8.2	*9.0*	7.5
Bisoprolol	**43**	Antagonist^17^	Antagonist^2^		Antagonist^2^	*7.7*	5.9	5.0
BRL 37344	**45**	Agonist/partial agonist^17^	Agonist/partial agonist^17^		Agonist/partial-full agonist^17^	5.2	5.8	6.5
Broxaterol	**48**	Antagonist^2^	Agonist/partial agonist^2^		Agonist^2^	5.9	5.9	5.4
Bunolol	**50**	Antagonist^17^	Antagonist^17^		Antagonist^17^	8.4	9.3	6.8
Bupranolol	**51**	Antagonist^17^	Antagonist^17^		Antagonist^17^	8.2	9.1	7.1
Carvedilol	**55**	Antagonist^17^	Antagonist^17^		Antagonist^17^	*9.0*	*9.3*	*7.7*
CGP 12177	**56**	Agonist/partial agonist; antagonist^17^	Antagonist^17^		Agonist/partial agonist^17^	*8.9*	*8.8*	*6.9*
Epinephrine ((−)-adrenaline)	**70**	Agonist^17^	Agonist/full agonist		Agonist/full agonist^17^	5.5	5.9	Inactive
Fenoterol (±)	**75**	Agonist^17^	Agonist^17^		Agonist/full agonist^17^	5.0	*5.9*	*5.4*
Formoterol (±)	**80**	Agonist^17^	Agonist^17^		Agonist/full agonist^43^	*6.2*	5.9	5.4
ICI 118, 551	**83**	Antagonist^2^	Antagonist/inverse agonist^17^		Antagonist/inverse agonist^2^	7.0	*9.3*	*6.2*
Indacaterol (R)	**86**	Agonist^17^	Agonist^17^		Agonist/full agonist^43^	*7.5*	*7.9*	8.3
isoprenaline	**87**	Agonist/full agonist^17^	Agonist/full agonist^17^		Agonist/full agonist^17^	6.6	6.5	5.7
Isoproterenol (+) (isoproterenol-l)	**89**	Antagonist^2^	Agonist/partial agonist^2^		Agonist^2^	6.6	6.3	5.8
Nadolol	**114**	Antagonist^17^	Antagonist^17^		Antagonist^17^	6.9	7.8	6.3
Nebivolol	**119**	Antagonist^17^	Antagonist^17^		Antagonist^17^	9.1	8.0	6.4
Noradrenaline- (−) (norepinephrine- (−))	**123**	Agonist^17^	Agonist^17^		Agonist/full agonist^17^	*5.6*	Inactive	*6.1*
Pindolol (±)	**128**	Antagonist; agonist/partial agonist	Antagonist^17^		Antagonist; partial agonist	*8.9*	*8.9*	*7.2*
Propranolol l-	**135**	Antagonist^17^	Antagonist^50^		Antagonist^2^	*8.6*	9.1	6.7
Salbutamol (±) (albuterol)	**140**	Antagonist^2^	Agonist/partial agonist^17^		Agonist^2^	5.9	*5.8*	8.3
Salmeterol	**141**	Antagonist/inverse agonist^2^	Agonist/partial agonist^17^		Antagonist/inverse agonist^2^	6.1	*6.9*	5.4
SR 59230A	**146**	Antagonist^17^	Antagonist^17^		Antagonist^17^	*7.9*	*8.1*	*7.3*

Merged binding affinities are written in italic. Agonists are green-colored, and antagonists and inverse agonists are orange-colored. If the ligand is characterized with both agonistic and antagonistic behavior, it is colored yellow ligands are sorted in ascending order of their ligand no. (highlighted in bold).

It is important to note that some ligands can act as agonist and antagonist at the same h*β*_x_AR, as described earlier. In general, the assignment of agonist properties (eg, partial vs full agonist) is context-dependent and can vary with receptor-coupled G-proteins and signaling pathways, assay conditions, experimental systems, and GTP concentrations.^2^^,^^7^^,^^9^^,^^66^ Although it has been reported that species-differences may play a minor role,^2^ the characterization is only valid for recombinant human systems. As a result, functional labels should be interpreted as conditional descriptions within a specific experimental context rather than as static properties of a ligand.^7^^,^^66^

**Fig. 4:**
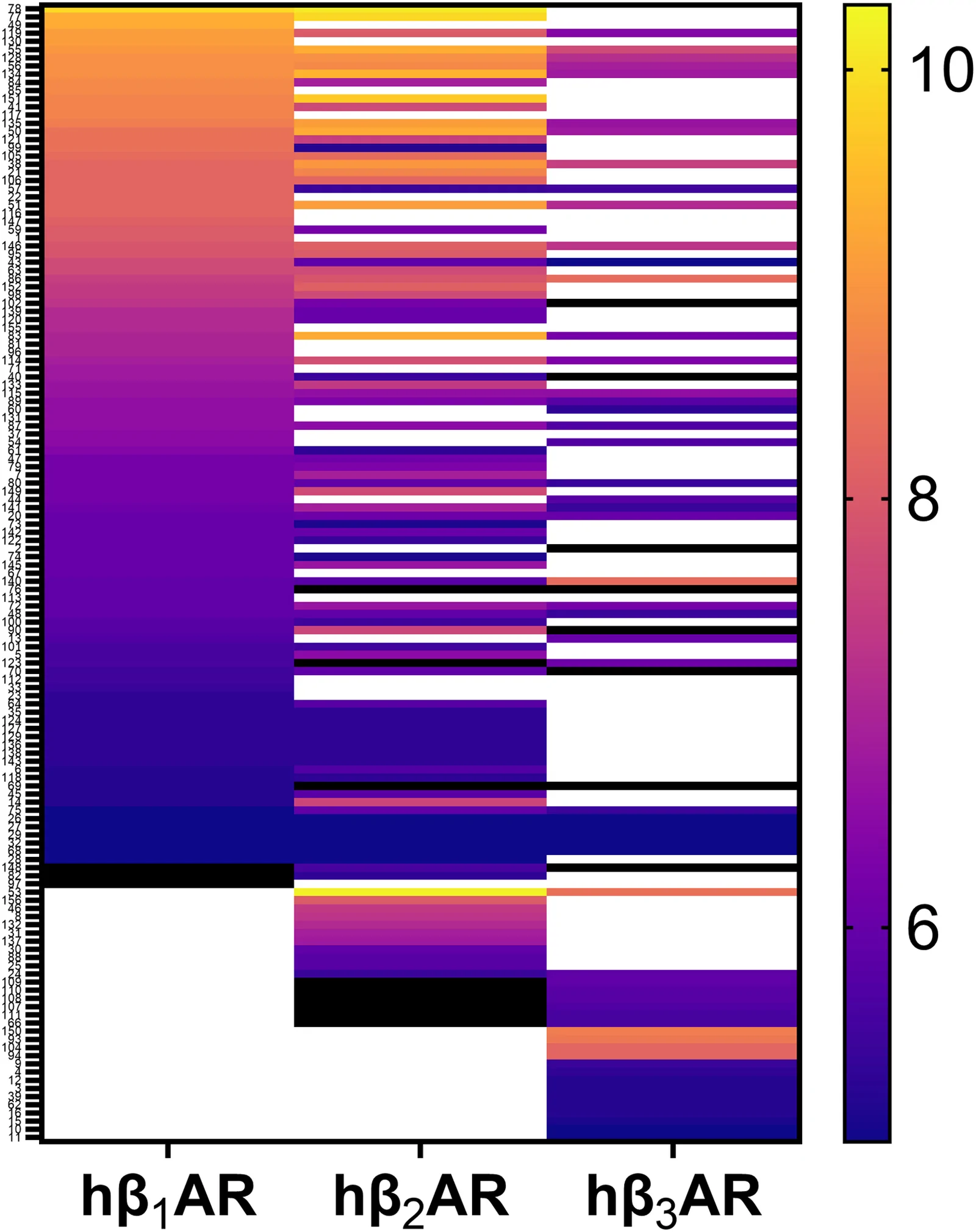
Heat map of binding affinities for all ligands, sorted in descending order of the highest binding affinity on the h*β*_1_AR (followed by the h*β*_2_AR and h*β*_3_AR). The heat map displays binding affinities (pK_i_ values) of all ligands identified in the PDSP K_i_ database for h*β*_x_AR. Each row corresponds to a single ligand, and each column represents one h*β*_x_AR. The numerical labels shown on the left side of the heat map correspond to the assigned ligand no. Binding affinity is visualized by color intensity according to the scale shown on the right, with higher pK_i_ values (stronger binding) indicated by yellow and lower pK_i_ values by blue. White areas show missing binding affinity data, and black areas highlight inactive binding (defined by the binding affinity threshold K_i_ exceeding 10,000 nM). Ligands are vertically ordered based on their highest reported binding affinity at h*β*_1_AR, with ligands showing the strongest h*β*_1_AR binding positioned at the top of the heat map and decreasing affinity toward the bottom. This sorting facilitates direct comparison of ligand binding profiles across receptor subtypes and allows visual identification of ligands with selective, partially overlapping, or broader binding affinity patterns. Differences in color intensity across columns within the same row reflect variations in binding affinity of individual ligands between h*β*_x_AR subtypes. Continuous color gradients indicate receptors with extensive receptor binding coverage, whereas fragmented or sparse patterns highlight incomplete experimental characterization or selective binding. Overall, the heat map illustrates that many ligands with high h*β*_1_AR affinity also binding actively to other h*β*_x_AR subtypes, particularly h*β*_2_AR.

Heat maps are presented in Fig. 4 and Supplemental Figs. 2 and 3, with color intensity reflecting binding strength and ligands ordered by affinity at a given h*β*_x_AR. Overall, h*β*_1_AR and h*β*_2_AR share a moderate degree of similarity, as several ligands with higher affinity at h*β*_1_AR also display measurable binding at h*β*_2_AR, and vice versa. In contrast, h*β*_3_AR shows a more distinct affinity pattern, characterized by clusters of ligands with high h*β*_3_AR selectivity and varying affinity at h*β*_1_AR and h*β*_2_AR.

## 6 Characteristics of clinically important h*β*_x_AR ligands

**Fig. 5:**
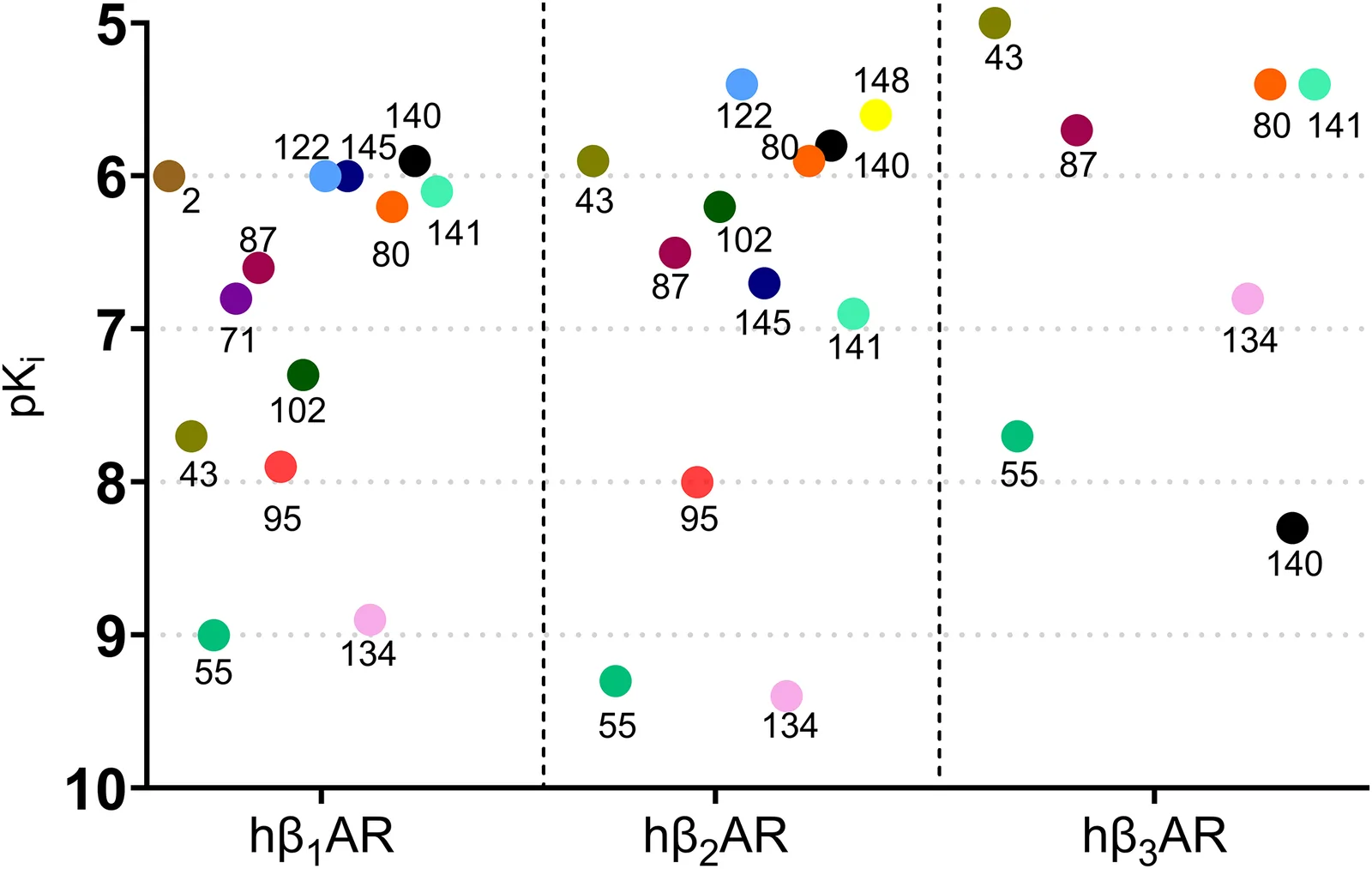
Overview about the binding affinities of clinically important ligands (Table 1) on h*β*_x_AR. Each compound is depicted as a colored dot and labeled with its ligand no. The following drugs are depicted: adrenaline (**2**), bisoprolol (**43**), carvedilol (**55**), esmolol (**71**), isoprenaline (**87**), labetalol (**95**), metoprolol (**102**), noradrenaline (**122**), sotalol (**145**), propranolol (**134**), formoterol (**80**), salbutamol (**140**), salmeterol (**141**), and terbutaline (**148**).

To relate binding affinity data to therapeutic use, clinically established h*β*_x_AR ligands were evaluated with respect to their subtype preference, functional classification, and reported adverse effect profiles.^2^^,^^3^^,^^6^^,^^17^ Table 5 provides a comprehensible overview of characterization and literature comparison for presented clinically important ligands. Table 6 connects these ligands with their pK_i_ values at h*β*_1_AR– h*β*_3_AR, IUPHAR-listed target receptors, therapeutic effects, and receptor-associated adverse effects. Additionally, they were visualized separately in Fig. 5 to enable an overview about affinity-based receptor profiles. This is particularly relevant because clinical labels such as “h*β*_1_AR-selective antagonist” or “h*β*_2_AR-selective agonist” may suggest a degree of receptor specificity that is not always reflected by binding affinity data.

**Table 5:** Overview and comparison of clinically important ligands of the summarized database-derived information (PDSP 2026; IUPHAR 2026) and relevant literature^2^^,^^3^

Drug	Ligand No.	Description^3^			Characterization PDSP K_i_ Database and IUPHAR/BPS Guide to Pharmacology^16,17^			Binding Affinity (pK_i_) PDSP K_i_ Database and IUPHAR/BPS Guide to Pharmacology^16,17^			Binding Affinity (pK_i_)^2^		
		h*β*_x_AR Target	Action	Properties	h*β*_1_AR	h*β*_2_AR	h*β*_3_AR	h*β*_1_AR	h*β*_2_AR	h*β*_3_AR	h*β*_1_AR	h*β*_2_AR	h*β*_3_AR
Adrenaline	**2**	1, 2, 3	Agonist	Nonselective, full agonist	**Agonist/full agonist****^17^**		**Agonist/full agonist****^17^**	**6.0**		**Inactive**	**5.4**	**6.1**	**Inactive**
Bisoprolol	**43**	1	Antagonist	Selective	**Antagonist****^17^**	Antagonist^2^	Antagonist^2^	7.7	5.9	5.0	**7.6**	5.9	5.0
Carvedilol	**55**	1, 2	Antagonist	Nonselective	**Antagonist****^17^**	**Antagonist****^17^**	Antagonist^17^	**9.0**	**9.3**	7.7	**8.8**	**9.0**	6.6
Esmolol	**71**	1	Antagonist	Short-acting, esterase-sensitive	**Antagonist****^17^**			**6.8**			**No data**	No data	No data
Formoterol	**80**	2	Agonist	Selective	Agonist^17^	**Agonist****^17^**	Agonist/full agonist^43^	6.2	**5.9**	5.4	5.8	**5.6**	5.1
Isoprenaline	**87**	1, 2	Agonist	Nonselective, full agonist	**Agonist/full agonist****^17^**	**Agonist/full agonist****^17^**	Agonist/full agonist^17^	**6.6**	**6.5**	5.7	**No data**	**No data**	No data
Labetalol	**95**	1, 2	Antagonist	Nonselective	**Antagonist****^17^**	**Agonist/partial agonist****^17^**		**7.9**	**8.0**		**No data**	**No data**	No data
Metoprolol	**102**	1	Antagonist	Selective	**Antagonist****^17^**	Antagonist^17^	Inactive (PDSP)	**7.3**	6.2	Inactive	**7.3**	5.5	5.0
Mirabegron	**103**	3	Agonist	Selective	Agonist^17^	Agonist^17^	**Agonist****^17^**	No data	No data	**No data**	No data	No data	**No data**
Noradrenaline	**122**	1, 2, 3	Agonist	Nonselective	**Agonist****^17^**	**Agonist/full agonist****^17^**		**6.0**	**5.4**		**5.4**	**Inactive**	**5.4**
Propranolol	**134**	1, 2, 3	Antagonist	Nonselective, high affinity	**Antagonist (PubChem 2026)**	**Antagonist****^17^**	**Antagonist****^17^**	**8.9**	**9.4**	**6.8**	**8.7**	**9.1**	**6.7**
Salbutamol	**140**	2	Agonist	Partial agonist	Antagonist^2^	**Agonist/partial agonist****^17^**	Agonist^2^	5.9	**5.8**	8.3	5.6	**5.7**	4.3
Salmeterol	**141**	2	Agonist	Selective	Antagonist/inverse agonist^2^	**Agonist/partial agonist****^17^**	Antagonist/inverse agonist^2^	6.1	**6.9**	5.4	5.8	**7.6**	5.1
Solabegron	**144**	3	Agonist	Selective	Agonist^17^	Agonist^17^	**Agonist****^17^**	No data	No data	**No data**	No data	No data	**No data**
Sotalol	**145**	1, 2	Antagonist	Nonselective	**Antagonist****^17^**	**Antagonist****^17^**		**6.0**	**6.7**		**No data**	**No data**	No data
Terbutaline	**148**	2	Agonist	Partial agonist	Agonist/partial agonist^17^	**Agonist/partial agonist****^17^**	Inactive (PDSP)	Inactive	**5.6**	Inactive	Inactive	**Inactive**	Inactive

Agonists are green-colored, and antagonists and inverse agonists are orange-colored. h*β*_x_AR targets described by Baker et al^3^ are highlighted in bold font for compared binding affinities and characterization. Ligands are sorted by their ligand no. (highlighted in bold).

**Table 6:** Discussion of therapeutic and adverse effects of clinically important ligands (Table 1) in relation to h*β*_x_AR binding affinities

Drug	Ligand No.	h*β*_1_AR pK_i_	h*β*_2_AR pK_i_	h*β*_3_AR pK_i_	Target Receptor^17^	Therapeutic Effect^3,20-22,24,25^	Adverse Effect and h*β*_x_AR Association^3,18,67,68^
Adrenaline (epinephrine)	**2**	6	No data	Inactive	h*β*_1_AR, h*β*_3_AR	Cardiac stimulation and bronchodilation	Tachycardia and arrhythmia mainly via h*β*_1_AR activation, systemic h*β*_x_AR activation due to lack of subtype selectivity
Bisoprolol	**43**	7.7	5.9	5	h*β*_1_AR	Reduction of cardiac sympathetic stimulation	Bradycardia via h*β*_1_AR blockade, bronchoconstriction possible through residual h*β*_2_AR affinity
Carvedilol	**55**	9	9.3	7.7	h*β*_1_AR, h*β*_2_AR	Broad h*β*_x_AR blockade with reduction of cardiac workload	Bronchoconstriction through h*β*_2_AR blockade, bradycardia via h*β*_1_AR blockade
Esmolol	**71**	6.8	No data	No data	h*β*_1_AR	Short-acting reduction of cardiac rate and conduction	Bradycardia and hypotension via h*β*_1_AR blockade; bronchoconstriction possible if h*β*_2_AR is affected
Formoterol	**80**	6.2	5.9	5.4	h*β*_1_AR, h*β*_2_AR	Bronchodilation via h*β*_2_AR activation	Tachycardia through h*β*_1_AR activation or incomplete h*β*_2_AR selectivity; tremor may result from h*β*_2_AR activation
Isoprenaline	**87**	6.6	6.5	5.7	h*β*_1_AR, h*β*_2_AR, h*β*_3_AR	Cardiac stimulation and bronchodilation	Tachycardia and arrhythmia mainly via h*β*_1_AR activation
Labetalol	**95**	7.9	8	No data	h*β*_1_AR, h*β*_2_AR	Reduction of cardiovascular sympathetic activity	Bronchoconstriction through h*β*_2_AR blockade; bradycardia via h*β*_1_AR blockade
Metoprolol	**102**	7.3	6.2	Inactive	h*β*_1_AR, h*β*_2_AR	Reduction of cardiac sympathetic stimulation	Bradycardia via h*β*_1_AR blockade; bronchoconstriction possible through residual h*β*_2_AR affinity
Mirabegron	**103**	No data∗	No data∗	No data∗	h*β*_1_AR, h*β*_2_AR, h*β*_3_AR	Detrusor relaxation via h*β*_3_AR activation	Cardiovascular adverse effects may occur if h*β*_1_AR or h*β*_2_AR are affected, although relevant activation is limited at therapeutic exposure
Noradrenaline	**122**	6	5.4	No data	h*β*_1_AR, h*β*_2_AR	Cardiac stimulation and systemic hemodynamic support	Tachycardia and arrhythmia mainly via h*β*_1_AR activation
Propranolol	**134**	8.9	9.4	6.8	h*β*_2_AR, h*β*_3_AR	Nonselective h*β*_x_AR blockade with cardiovascular and extracardiac effects	Bronchoconstriction through h*β*_2_AR blockade; bradycardia via h*β*_1_AR blockade
Salbutamol	**140**	5.9	5.8	8.3	h*β*_2_AR	Rapid bronchodilation via h*β*_2_AR activation	Tachycardia and cardiovascular adverse effects are pharmacologically plausible due to measurable h*β*_1_AR affinity and systemic h*β*_x_AR activation
Salmeterol	**141**	6.1	6.9	5.4	h*β*_2_AR	Long-acting bronchodilation via h*β*_2_AR activation	Tachycardia possible through incomplete h*β*_x_AR subtype selectivity or systemic exposure
Solabegron	**144**	No data∗	No data∗	No data∗	h*β*_1_AR, h*β*_2_AR, h*β*_3_AR	Detrusor relaxation via h*β*_3_AR activation	Cardiovascular adverse effects may occur if h*β*_1_AR or h*β*_2_AR are affected, although relevant activation is limited at therapeutic exposure
Sotalol	**145**	6	6.7	No data	h*β*_1_AR, h*β*_2_AR	Reduction of cardiac sympathetic stimulation	Bradycardia via h*β*_1_AR blockade; bronchoconstriction through h*β*_2_AR blockade
Terbutaline	**148**	Inactive	5.6	Inactive	h*β*_1_AR, h*β*_2_AR	Rapid bronchodilation via h*β*_2_AR activation	Tachycardia possible through incomplete receptor subtype selectivity or systemic exposure; tremor may result from h*β*_2_AR activation

Provided are the drug name, assigned ligand number, binding affinities at h*β*_1_AR – h*β*_3_AR, target receptors listed in the IUPHAR/BPS Guide to Pharmacology, therapeutic effect, and receptor-associated adverse effects. The therapeutically relevant effects are linked with the collected binding affinities (Table 2) and illustrates that additional h*β*_x_AR binding occurs beyond the listed therapeutic target receptor. Ligands are sorted by ligand no. (highlighted in bold). A visualization of the binding affinities of these ligands is provided in Fig. 5.

No data ∗ indicates that affinity data was available (pEC50), but not in the measure of the collected binding affinity (Ki); inactive implies pKi < 5.

The endogenous catecholamines adrenaline (**2**) and noradrenaline (**122**) are described as nonselective full agonists at h*β*_1_AR and h*β*_2_AR with lower activity at h*β*_3_AR (Table 5). This combination of nonselective h*β*_x_AR activation and full-agonistic property explains their rapid and pronounced cardiovascular and bronchial effects, including increased heart rate, myocardial contractility, and bronchodilation.^69^ The absence of subtype selectivity accounts for their efficacy in emergency indications such as shock and anaphylaxis by their systemic cardiovascular stimulation.^3^ Accordingly, their therapeutic effects can be assigned to broad h*β*_x_AR activation, whereas tachycardia and arrhythmia are mainly linked to h*β*_1_AR activation (Table 6).

h*β*_1_AR-preferring antagonists such as metoprolol (**102**) and bisoprolol (**43**) display higher affinity at h*β*_1_AR than at h*β*_2_AR, consistent with their classification as h*β*_1_AR-selective antagonists.^3^ However, the observed affinity differences correspond to relative h*β*_1_AR preference rather than absolute subtype selectivity, relating to higher affinity at h*β*_1_AR than at h*β*_2_AR or h*β*_3_AR, but not complete absence of affinity at other receptor subtypes (Fig. 5). Their therapeutic benefit in chronic cardiovascular disease derives from attenuation of h*β*_1_AR-mediated cardiac stimulation.^22^ Residual h*β*_2_AR affinity, although lower, provides a mechanistic basis for dose-dependent broncho-constrictive adverse effects, particularly in patients with present respiratory conditions.^18^^,^^67^ Thus, the desired therapeutic effects are mainly linked to h*β*_1_AR blockade, whereas residual h*β*_2_AR binding is assigned to possible respiratory adverse effects (Table 6).

Nonselective high affinity antagonists such as propranolol (**134**) and carvedilol (**55**) exhibit comparable and high pK_i_ values at h*β*_1_AR and h*β*_2_AR, with measurable affinity at h*β*_3_AR (Table 5; Fig. 5). Their lack of subtype discrimination corresponds to simultaneous blockade of cardiac and bronchial h*β*_x_AR. The high antagonistic affinity at both h*β*_1_AR and h*β*_2_AR explains their potent inhibitory effect but also the increased risk of bronchospasm and peripheral h*β*_2_AR-mediated adverse effects compared with h*β*_1_AR-selective drugs.^3^ Table 6 further illustrates that these ligands may show broader h*β*_x_AR binding than suggested by individual therapeutic h*β*_x_AR targets.

h*β*_2_AR agonists such as salbutamol (**140**), terbutaline (**148**), salmeterol (**141**), and formoterol (**80**) show moderate binding affinity, with variable h*β*_2_AR selectivity. They display receptor preference rather than absolute h*β*_2_AR selectivity (Fig. 5). Salbutamol and terbutaline are described as partial agonists at h*β*_2_AR. Their moderate affinity and partial intrinsic activity limit maximal receptor activation, which contributes to effective bronchodilation with comparatively reduced cardiac stimulation relative to full, nonselective agonists such as isoprenaline (**87**).^70^ Salmeterol (**141**) and formoterol (**80**) exert sustained effects, consistent with their long-acting broncho-dilatory effect.^3^^,^^23^^,^^26^ Nonetheless, incomplete subtype exclusivity and systemic exposure can result in h*β*_1_AR-mediated tachycardia at higher doses.^68^ This is especially relevant for salbutamol (**140**), which is commonly considered h*β*_2_AR-selective but still shows relevant affinity at h*β*_1_AR in the compiled data (Fig. 5). Therefore, the clinical occurrence of cardiovascular adverse effects during excessive exposure is pharmacologically plausible and consistent with incomplete receptor subtype discrimination. The desired broncho-dilatory effect is mainly due to h*β*_2_AR activation, whereas h*β*_1_AR affinity and systemic h*β*_x_AR activation are linked to cardiovascular adverse effects.

For h*β*_3_AR agonists such as mirabegron (**103**) and solabegron (**144**), functional classification consistently indicates agonist activity at h*β*_3_AR, whereas subtype affinity data remain limited. Therapeutic detrusor relaxation in overactive bladder corresponds to h*β*_3_AR activation. The absence of relevant h*β*_1_AR and h*β*_2_AR activation at therapeutic exposure is consistent with the low incidence of cardiovascular and respiratory adverse effects observed in clinical use.^71^^,^^72^

Across ligand classes, most compounds demonstrate subtype preference rather than strict selectivity at the affinity level. The combination of quantitative binding affinity and intrinsic efficacy (full vs partial agonism or antagonism) determines the extent and specificity of receptor-mediated effects. High affinity nonselective ligands produce broader effects, whereas subtype preference can limit but not eliminate off-target responses. Therefore, therapeutic receptor assignment should be interpreted as receptor preference rather than absolute selectivity, especially when binding affinity data reveal measurable activity at additional h*β*_x_AR (Table 6).

## 7 Comparison of stereoisomers

In the characterization of ligands, it is important to consider chirality. In the ligand overview of the PDSP K_i_ database and the IUPHAR/BPS Guide to Pharmacology, multiple stereoisomers are sometimes listed for a single ligand, with apparent differences in binding affinity at h*β*_x_AR. Since there is extensive evidence that stereoisomers of a substance can display marked differences in binding at h*β*_x_AR,^3^^,^^8^ binding affinities were compared whenever at least 2 stereoisomers or 1 stereoisomer and a racemate were available (Table 7).

**Table 7:** Stereoisomer comparison of ligands with available data for at least 2 stereoisomer variants for a specific h*β*_x_AR

Ligand Name	Ligand No.	Synonym	Stereoisomer	h*β*_1_AR pK_i_	h*β*_2_AR pK_i_	h*β*_3_AR pK_i_	h*β*_1_AR Comparison of Stereo-Isomers	h*β*_2_AR Comparison of Stereo-Isomers	h*β*_3_AR Comparison of Stereo-Isomers	h*β*_1_AR Stereo-Isomer With Highest pK_i_	h*β*_2_AR Stereo-Isomer With Highest pK_i_	h*β*_3_AR Stereo-Isomer With Highest pK_i_	Literature Comparison
Adrenaline	**70**	Epinephrine	-	5.2–6.0	5.7–6.2	3.9–4.7	0.0–0.8 ↓		0.0 →	racemate		racemate and (-)-stereoisomer	Consistent^8^
		Epinephrine	+	not listed	not listed	not listed							
	**2**	Epinephrine	+-/racemate	6	not listed	3.9–4.7	0.0–0.8↑		0.0 →				
Nor-adrenaline	**123**	Norepinephrine	-	5.5–6.0	4.6–5.4	4.7–6.6	0.0–0.5 ↓	0.0–0.8 ↓		racemate	racemate		Consistent^8^
		Norepinephrine	+	not listed	not listed	not listed							
	**122**		+-/racemate	6	5.4	not listed	0.0–0.5 ↑	0.0–0.8 ↑					
Salbutamol	**97**	levosalbutamol, levoalbuterol	R	4.7	not listed	not listed				racemate			Contradictory^73^
			S	not listed	not listed	not listed							
	**140**	albuterol	+-/racemate	5.9	5.3-6.1	8.3							
Propranolol	**135**		S	7.9–8.9	9.1	6.7	0.8 (↓) – 0.2 (↑)	0.0–0.4 ↓	0.5 (↓) – 0.4 (↑)	racemate	racemate	racemate	Contradictory^74^
		dextropropranolol	R	not listed	not listed	not listed							
	**134**		+-/racemate	8.7	9.1–9.5	6.3–7.2	0.2 (↓) – 0.8 (↑)	0.0–0.4 ↑	0.4 (↓) – 0.5 (↑)				
Fluoro-carazolol	**77**		R	9.3	9.9	not listed	0.6 ↓	0.2 ↓		S-stereoisomer	S-stereoisomer		No literature found
	**78**		S	9.9	10.1	not listed	0.6 ↑	0.2 ↑					
			+-/racemate	not listed	not listed	not listed							
Iso-proterenol	**88**		-	not listed	5.8	not listed		0.5-0.8 ↓		(+)-stereoisomer	racemate	(+)-stereoisomer	Contradictory^75^
	**89**	Isoproterenol-I	+	6.6	6.3	5.8	0.4 (↓) – 0.5 (↑)	0.3 (↓) – 0.5 (↑)	0.5–1.6 ↑				
		isoprenaline	+-/racemate	6.1–7.0	6.4–6.6	4.2–5.3	0.5 (↓) – 0.4 (↑)	0.1–0.8 ↑	0.5–1.6 ↓				
Fen-fluramine	**74**		R/-	6.0	5.1	not listed	→	→			racemate and (-)-stereoisomer	racemate and (-)-stereoisomer	No literature found
			S/+	inactive	inactive	not listed	↓	↓					
	**73**		+-/racemate	6.0	5.1	not listed	→	→					

Each compared pair is colored in a similar color, with arrows indicating whether the binding affinity is higher or lower than another stereoisomer of the same ligand. Furthermore, the superior ligand is indicated and a comparison with literature is provided to whether the findings are consistent (green-colored), contradictory (orange-colored), or if no information was found (gray -colored). Ligand no. are highlighted in bold.

Notably, the data are highly incomplete. For most ligands, no data for additional stereoisomers were available. For 5 ligands, data were found for either the racemate and the (−)- stereoisomer (adrenaline (**2, 70**), noradrenaline (**122, 123**), propranolol (**134, 135**)) or the (R)- and (S)- stereoisomer (fluorocarazolol (**77, 78**)). Only for 2 ligands (isoproterenol (**88, 89**), fenfluramine (**73, 74**)), binding affinities were available for the racemate as well as the (−)- and (+)-stereoisomer. For no ligand, data were available for all stereoisomers across all 3 h*β*_x_AR.

The highest binding affinity in available data was observed for the racemate of propranolol (**134, 135**) (all 3 h*β*_x_AR), noradrenaline (**97, 122**) (h*β*_1_AR and h*β*_2_AR), and salbutamol (**135, 140**) (h*β*_1_AR). In fluorocarazolol (**77, 78**), the highest binding affinity was observed for the S-stereoisomer (h*β*_1_AR and h*β*_2_AR) although no data were available for the racemate.

A variable pattern of superiority in binding affinities was found for isoproterenol (**88, 89**), where the racemate has the highest pK_i_ value for h*β*_2_AR, whereas the (+)-stereoisomer was superior for h*β*_1_AR and h*β*_3_AR. Comparable patterns were observed in fenfluramine (**73, 74**), with similar binding affinities for the racemate and (−)-stereoisomer in h*β*_2_AR and h*β*_3_AR, whereas the (+)-stereoisomer is reported to be inactive on both h*β*_x_AR. For adrenaline (**2, 70**), the racemate and (−)-stereoisomer showed similar binding on the h*β*_3_AR, whereas the racemate was superior on the h*β*_1_AR.

It is not possible to conclusively identify the eutomer (higher activity) or distomer (lower activity)^8^ in a systematic manner across h*β*_x_AR because of the incomplete availability of binding affinities across stereoisomers and h*β*_x_AR. It may be assumed that some stereoisomers are not listed for recombinant h*β*_x_AR because of inactive binding in initial experimental investigations. However, this assumption cannot be confirmed, given the generally fragmented affinity data in both databases. Only if a stereoisomer is explicitly reported as inactive (eg, the (+)-stereoisomer of fenfluramine (**73, 74**)), this can be concluded. Regarding ΔpK_i_, absolute differences between stereoisomers are often within one log. However, in some of the examined ligands, absolute differences in binding affinities exceed one log, which implies a certain degree of receptor subtype selectivity within stereoisomers. This includes isoproterenol (**88, 89)** (Δh*β*_3_AR 0.5–1.6) and fenfluramine (**73, 74**) (Δh*β*_1_AR > 1.0) (Table 7).

Although literature confirms the found pattern of superiority of the (−)-stereoisomer for adrenaline (**2, 70**) and noradrenaline (**122, 123**),^8^ conclusions regarding many other ligands are contradicting. For salbutamol (**97, 140**), available binding affinity data indicates a higher binding affinity of the racemic salbutamol over the (R)-stereoisomer, whereas literature reports a much higher efficacy of salbutamol-(R) over –(S) and the racemate.^73^ In isoproterenol, it is reported that the (−)-stereoisomer is more potent than the (+)-stereoisomer75, whereas the comparison of binding affinity data shows a superiority of the (+)- and racemate, with data for the (−)-stereoisomer only being available for the h*β*_2_AR. Similar issues are reported for propranolol (**134, 135**), whereas the racemate was found to be slightly more potent in the available data, literature reports the (S)-stereoisomer to be more potent than the (R)-stereoisomer, for which no binding affinity data were available in the databases.^74^

Overall, the present dataset does not allow identification of stereoisomeric superiority across h*β*_x_AR. Although the racemate frequently displayed the highest binding affinity within the limited available data, this observation likely results from incomplete stereoisomer coverage and issues from the interpretation of absolute binding values from different methodological approaches. Consequently, ligand comparisons are sometimes contradicted by literature findings, and no robust general tendencies could be derived from the present comparison. However, literature describes certain recurring patterns. In general, it is considered that the (−)-stereoisomer is more potent than the (+)-stereoisomer, with confirming evidence for catecholamines, for example, adrenaline (**2, 70**) and noradrenaline (**122, 123**).^8^^,^^12^ This highlights that individual stereoisomers may differ substantially from the racemic mixture. For fenoterol (**75**), the IUPHAR/BPS Guide to Pharmacology states for that the displayed racemate is much less active than the (S, S)- and (R, R)-stereoisomers,^17^ with more complex differences and selectivity across multiple stereoisomer centers.^13^

As discussed earlier, receptor preference depends on the coupled G protein, analyzed receptor system and parameters.^3^^,^^8^ Binding affinity therefore represents a context-dependent parameter rather than an absolute measure of stereoisomeric superiority. The incomplete and partly contradictory findings of the present comparison further emphasize that absolute binding affinity values must be interpreted cautiously and in relation to experimental context.

## 8 Comparison of ligand and binding affinity listings between the PDSP K_i_ database and the IUPHAR/BPS Guide to Pharmacology

Large differences are observed between the PDSP K_i_ database and the IUPHAR/BPS Guide to Pharmacology with regard to the available ligands and binding affinities for h*β*_x_AR. Both sources list a comparable number of ligands (IUPHAR/BPS *n* = 105; PDSP *n* = 96). Notably, only a small number of ligands are listed in both databases (*n* = 45), indicating low agreement (28.8%). The majority of ligands (*n* = 111, 71.2%) are listed in only 1 of the 2 databases.

This low agreement in ligand listings is also reflected in the small number of binding affinities for the same ligand available in both databases (*n* = 42, 15.4%), compared with a large number of pK_i_ values only found in only 1 database (*n* = 231, 84.6%).

A considerable number of ligands is omitted in each database (Supplemental Table 4). For the PDSP K_i_ database, no binding affinity data (including no “inactive” binding) are found for 51 ligands that are listed in the other database. Analog, in the ligand search of the IUPHAR/BPS Guide to Pharmacology, the respective ligand is not listed (*n* = 60). In addition, ligands are often found in the IUPHAR/BPS ligand search but without an assigned *β*_x_AR target (*n* = 74).

Incomplete binding affinity data are also observed. In some cases, pK_i_ values for certain h*β*_x_AR subtypes are available for a given ligand in one database, whereas active binding affinity data for further h*β*_x_AR are found only in the complementary database. This is observed in the PDSP Ki database for 2 ligands (BRL 37344 (**45**), epinephrine (**70**)), and in the IUPHAR/BPS Guide to Pharmacology for 8 ligands (formoterol (±) (**80**), ICI 118, 551 (**83**), indacaterol (R) (**86**), salbutamol (±) (**140**), salmeterol (**141**), alprenolol (±) (**38**), bisoprolol (**43**), propranolol (I-) (**135**)). Taken together, these findings highlight considerable divergence in both ligand listings and the availability of binding affinity data across the 2 databases.

Besides the varying ligand listings, both the PDSP K_i_ database and the IUPHAR/BPS Guide to Pharmacology contain a range of binding affinities for duplicate entries, as well as deviations in pK_i_ for ligands listed in both databases. In the PDSP K_i_ database, the range of binding affinities among duplicates is 0.0 to 3.2 pK_i_ (h*β*_1_AR 0.0–2.7, h*β*_2_AR 0.0–2.5, and h*β*_3_AR 0.0–3.2). In the IUPHAR/BPS Guide to Pharmacology, the corresponding range is 0.0–1.6 pK_i_ (h*β*_1_AR 0.0–1.1, h*β*_2_AR 0.1–1.6, and h*β*_3_AR 0.1–1.3). When comparing similar ligand entries available in both databases, the variation in pK_i_ values is comparable the duplicate ranges (h*β*_1_AR 0.2–1.5, h*β*_2_AR 0.1–1.7, and h*β*_3_AR 0.0–2.2).

## 9 Current challenges and knowledge gaps

The analysis of ligands and binding affinities is strongly dependent on the data sources PDSP K_i_ database, IUPHAR/BPS Guide to Pharmacology, and the experimental data in the underlying primary literature. Methodology differences may affect accurateness and comparability of ligand profiling, including binding affinities and functional characterization. The observed deviations and ranges in pK_i_ values therefore limit precision, and functional labels may be outdated, inconsistently defined, or dependent on assay conditions.

Another important restriction is the initial selection of ligands with binding affinities below 10,000 nM. Although this inclusion criterion is widely applied and necessary to filter relevant data, it may limit the completeness of the characterization of ligands. In combination with incomplete receptor profiling, this threshold further restricts the ability to draw definitive conclusions regarding receptor selectivity for many ligands. It is not always possible to clearly identify highly selective behavior if the ligand has not been examined on all h*β*_x_AR or if there are duplicates available with a wide pK_i_ range.

Finally, the stereoisomer analysis is limited by incomplete and heterogeneous data. For no ligand, all stereoisomers and h*β*_x_AR were available, and methodological differences may account for discrepancies between database-derived affinities and literature-reported potency. Consequently, conclusions regarding stereoisomeric superiority and absolute binding affinity are restrained.

## 10 Conclusions and future perspectives

The analysis provides a structured overview of ligands acting at recombinant h*β*_x_AR and demonstrates that a considerable number of compounds exhibit active binding (Table 2). These ligands can broadly be categorized as either subtype-selective or as displaying broader multireceptor activity (Figs. 3 and 4; Supplemental Figs. 2 and 3). A pronounced similarity between h*β*_1_AR and h*β*_2_AR is reflected in their overlapping pK_i_ distributions, median affinities, and frequent shared ligand profiles, which is mechanistically concordant with their high structural homology in ligand binding pockets.^3^^,^^27^ In contrast, h*β*_3_AR appears less extensively investigated and pharmacologically more distinct,^76^ which is evident from its narrower data coverage and more confined affinity range. The clinical relevance of h*β*_1_AR and h*β*_2_AR in cardiovascular and respiratory therapy is mirrored by the higher density of available ligands and functional annotations for these subtypes.

The characterization of clinically important ligands further demonstrates that therapeutic subtype classification does not necessarily correspond to strict affinity-based selectivity. This is particularly relevant for drugs such as salbutamol (**140**), which are commonly regarded as h*β*_2_AR-selective but nevertheless show measurable h*β*_1_AR and h*β*_3_AR affinity in the compiled data (Table 1; Fig. 5). The comparison of IUPHAR/BPS-listed target h*β*_x_AR, compiled pK_i_ values, therapeutic effects and receptor-associated adverse effects further indicates that established therapeutic target assignment does not exclude additional receptor binding (Table 6). Thus, clinically relevant adverse effects may partly arise from the incomplete subtype discrimination that is visible at the binding affinity level.

In general, the dataset must be interpreted as a relative rather than absolute representation of ligand–receptor interactions. Many ligands lack complete functional characterization across all 3 h*β*_x_AR. The comparison between the PDSP K_i_ database and the IUPHAR/BPS Guide to Pharmacology reveals substantial differences in ligand listings and availability of binding data. The low overlap between databases and the variability of duplicate entries underscore the fragmented nature of currently accessible affinity data. Consequently, apparent selectivity or potency may partly reflect incomplete reporting rather than true pharmacological specificity.

A central finding of this work is that binding affinity values are highly context-dependent. Experimental systems, including receptor expression systems, assay conditions, and data evaluation procedures, substantially influence pK_i_ values. Of particular importance are the chosen coupled G-proteins, intracellular signaling context, and the presence or absence of GTP.^2^^,^^3^^,^^7^^,^^8^ GTP modulates receptor–G protein coupling and can shift receptors between high- and low affinity states, thereby directly affecting measured binding affinity.^9^ Likewise, different G protein coupling profiles can alter the apparent efficacy and even the qualitative functional classification of a ligand.^2^ The observed inconsistencies in agonist, antagonist, and inverse agonist labeling across studies^17^ illustrate that functional behavior is not an intrinsic and stable ligand characteristic, but rather a system-dependent outcome.

The stereoisomer comparison further highlights these limitations. Incomplete stereochemical coverage and heterogeneous methodological backgrounds prevent robust conclusions regarding stereoisomeric superiority across h*β*_x_AR. In several cases, database-derived affinity data diverge from literature reports on potency or efficacy. These findings emphasize that absolute pK_i_ values, particularly when extracted from different sources and methodologies, should be interpreted cautiously and set in relation to their experimental context.

Overall, this analysis demonstrates both the value and the limitations of integrating fragmented pharmacological data. Although the compiled overview enables identification of broad binding patterns, subtype similarities, and potential selectivity trends, it also exposes significant gaps in ligand characterization, particularly for h*β*_3_AR, and inconsistencies in functional annotation. Future research should prioritize systematic comparative studies under standardized experimental conditions, including defined G protein coupling states and controlled GTP conditions, as well as comprehensive stereoisomer profiling.

The approach presented here offers a framework for structured cross-database ligand analysis and can be extended to other GPCR families. Nevertheless, any interpretation of binding affinity data must remain cautious and explicitly acknowledge its methodological relativity. Only through standardized and consistent experimental conditions, and transparent reporting, more reliable conclusions regarding ligand selectivity, efficacy, and therapeutic relevance can be achieved.

## Conflict of interest

The authors declare no conflicts of interest.
