# Supplementary material for: Comparative analysis of ligand binding affinities and functional profiles at recombinant human β-adrenergic receptor subtypes 1, 2, and 3
Source: J Pharmacol Exp Ther. 2026 Jun 8;393(7):104960. doi: 10.1016/j.jpet.2026.104960 (PMC13494184; doi:10.1016/j.jpet.2026.104960)
Supplement: Supplementary file 1 [file mmc1.docx]

**Comparative analysis of ligand binding affinities and functional profiles at recombinant human**

**β-adrenergic receptor subtypes 1, 2 and 3**

**– Supplement**

**Lilly Josephine Bindel and Roland Seifert**

Supplemental Figures

***Supplemental Figure 1:*** *Distribution (%) of binding affinities (pK_i_) of ligands at hβ_x_AR. Separate panels are shown for a) hβ_1_AR, b) hβ_2_AR, and c) hβ_3_AR. Black-colored bars represent the relative number of ligands per binding affinity value (pK_i_).*

***Supplemental Figure 2:*** *Heat map of binding affinities for all ligands, sorted in descending order of the highest binding affinity on the hβ_2_AR (followed by the hβ_1_AR and hβ_3_AR).*

*The heat map displays binding affinities (pK_i_ values) of all ligands identified in the PDSP K_i_ database for hβ_x_AR. Each row corresponds to a single ligand, and each column represents one hβ_x_AR. The numerical labels shown on the left side of the heat map correspond to the assigned ligand no. Binding affinity is visualized by color intensity according to the scale shown on the right, with higher pK_i_ values (stronger binding) indicated by yellow and lower pK_i_ values by blue. White areas show missing binding affinity data, and black areas highlight inactive binding (defined by the binding affinity threshold K_i_ exceeding 10,000 nM). Ligands are vertically ordered based on their highest reported binding affinity at hβ_1_AR, with ligands showing the strongest hβ_2_AR binding positioned at the top of the heat map and decreasing affinity towards the bottom. This sorting facilitates direct comparison of ligand binding profiles across receptor subtypes and allows visual identification of ligands with selective, partially overlapping, or broader binding affinity patterns. Differences in color intensity across columns within the same row reflect variations in binding affinity of individual ligands between hβ_x_AR subtypes. Continuous color gradients indicate receptors with extensive receptor binding coverage, whereas fragmented or sparse patterns highlight incomplete experimental characterization or selective binding.* *Overall, the heat map illustrates that many ligands with high hβ_2_AR affinity also binding actively to other hβ_x_AR subtypes, particularly hβ_1_AR.*

***Supplemental Figure 3:*** *Heat map of binding affinities for all ligands, sorted in descending order of the highest binding affinity on the hβ_3_AR (followed by the hβ_1_AR and hβ_2_AR).*

*The heat map displays binding affinities (pK_i_ values) of all ligands identified in the PDSP K_i_ database for hβ_x_AR. Each row corresponds to a single ligand, and each column represents one hβ_x_AR. The numerical labels shown on the left side of the heat map correspond to the assigned ligand no. Binding affinity is visualized by color intensity according to the scale shown on the right, with higher pK_i_ values (stronger binding) indicated by yellow and lower pK_i_ values by blue. White areas show missing binding affinity data, and black areas highlight inactive binding (defined by the binding affinity threshold K_i_ exceeding 10,000 nM). Ligands are vertically ordered based on their highest reported binding affinity at hβ_1_AR, with ligands showing the strongest hβ_2_AR binding positioned at the top of the heat map and decreasing affinity towards the bottom. This sorting facilitates direct comparison of ligand binding profiles across receptor subtypes and allows visual identification of ligands with selective, partially overlapping, or broader binding affinity patterns. Differences in color intensity across columns within the same row reflect variations in binding affinity of individual ligands between hβ_x_AR subtypes. Continuous color gradients indicate receptors with extensive receptor binding coverage, whereas fragmented or sparse patterns highlight incomplete experimental characterization or selective binding.* *Overall, the heat map illustrates that many ligands with high hβ_2_AR affinity also binding actively to other hβ_x_AR subtypes, but with a more distinct pattern.*

Supplemental Tables

***Supplemental Table 1:*** *Overview of frequently used radioligands for the determination of binding affinities on hβ_x_AR. Information is provided about the radioligand, exemplary compounds analyzed and reported pK_d_ values. pK_d_ represents the negative decadic logarithm of the equilibrium dissociation constant of the radioligand. Higher pK_d_ values correspond to higher radioligand affinity, whereas lower pK_d_ values indicate lower affinity.*

| **Radioligand** | **Exemplary compounds analyzed [14]** | **hβ_1_AR pK_d_ [15]** | **hβ_2_AR pK_d_ [15]** | **hβ_3_AR pK_d_ [15]** |
| --- | --- | --- | --- | --- |
| [^125^I]CYP ([^125^I]cyanopindolol) | propranolol (**134**),  pindolol (+/-) (**128**),  atenolol (**40**), epinephrine (**70**) | 10.4-11.3 | 11.1-11-4 | 9.2-9.8 |
| [^3^H]CGP 12177 | lisuride (**98**), bromocriptine (**47**), terguride (**149**) | - | 9.8 | 7.0 |
| [^3^H]DHA ([^3^H]dihydroalprenolol) | mCPP (**101**), olanzapine (**124**) | - | 10.1 | - |

***Supplemental Table 2:*** *Overview about the number and share of ligands, as well as the binding affinity (pK_i_) median, range, minimum and maximum for all three hβ_x_AR.*

| **hβ_x_AR** | **No. of ligands with active binding** | **Share of ligands** | **pK_i_ median** | **pK_i_ range** | **pK_i_ max** | **pK_i_ min** |
| --- | --- | --- | --- | --- | --- | --- |
| *hβ1AR* | 115 | 73.7% | 6.55 | 4.9 | 9.9 | 5.0 |
| *hβ_2_AR* | 105 | 67.3% | 6.15 | 5.2 | 10.2 | 5.0 |
| *hβ3AR* | 47 | 30.1% | 5.80 | 3.6 | 8.6 | 5.0 |
| Sum of binding affinities | 267 |  |  |  |  |  |
| Sum of ligands | 156 |  |  |  |  |  |

***Supplemental Table 3:*** *Overview of ligands considered as subtype-selective (ΔpK_i_ > 1) in the comparison of hβ_1_AR and hβ_2_AR. Only ligands with available binding affinity data for both receptor subtypes were included. Selectivity is defined as a difference in binding affinity of at least one logarithmic unit (ΔpK_i_ ≥ 1), corresponding to a ≥10-fold difference in Ki values. The table indicates the preferential receptor subtype (hβ_1_AR or hβ_2_AR) and the corresponding ΔpK_i_ values, based on the merged binding affinities presented in Table 1. Ligands are sorted by preferential receptor subtype and ligand number.*

| **Ligand** | **Ligand no.** | **Selectivity** | **ΔpK_i_** | **hβ_1_AR pK_i_** | **hβ_2_AR pK_i_** |
| --- | --- | --- | --- | --- | --- |
| Atenolol | **40** | hβ_1_AR | 1.3 | 6.8 | 5.4 |
| Bisoprolol | **43** | hβ_1_AR | 1.8 | 7.7 | 5.9 |
| CGP 20712-A | **57** | hβ_1_AR | 2.8 | 8.2 | 5.4 |
| Cicloprolol | **59** | hβ_1_AR | 1.8 | 8.0 | 6.2 |
| Cis-2a | **61** | hβ_1_AR | 1.1 | 6.4 | 5.3 |
| ICI 89, 406 | **84** | hβ_1_AR | 1.9 | 8.8 | 6.9 |
| LK 204-545 | **99** | hβ_1_AR | 3.2 | 8.4 | 5.2 |
| Metoprolol (+/-) | **102** | hβ_1_AR | 1.1 | 7.3 | 6.2 |
| Nebivolol | **119** | hβ_1_AR | 1.2 | 9.1 | 8.0 |
| NIHP (1-[4-(3-hydroxypropyl)phenoxy]-3-(propan-2-ylamino)propan-2-ol) | **120** | hβ_1_AR | 1.1 | 7.1 | 6.0 |
| S S-2c | **139** | hβ_1_AR | 1.1 | 7.1 | 6.0 |
| (Â±)-4-Ethylthio-2 5-dimethoxyamphetamine | **14** | hβ_2_AR | 2.4 | 5.2 | 7.6 |
| ICI 118, 551 | **83** | hβ_2_AR | 2.2 | 7.0 | 9.3 |
| KUR-1246 | **90** | hβ_2_AR | 1.8 | 5.8 | 7.6 |
| Terguride | **149** | hβ_2_AR | 1.5 | 6.2 | 7.7 |
| Timolol | **151** | hβ_2_AR | 1.1 | 8.7 | 9.7 |

***Supplemental Table 4:*** *Comparison of listed ligands for hβ_x_AR between the PDSP K_i_ database and the IUPHAR/BPS Guide to Pharmacology [14, 15].*

| **All three hβ_x_AR listed in both databases (n=12)** | **Ligand listed but binding affinities for at least one hβ_x_AR missing (n=10)** | **Ligand listed in IUPHAR/BPS Guide to Pharmacology but hβ_x_AR target omitted (n=23)** | **Ligand omitted in PDSP K_i_ database (n=60)** | **Ligand omitted in IUPHAR/BPS Guide to Pharmacology (n=51)** |
| --- | --- | --- | --- | --- |
| Carvedilol | BRL 37344 | (Â±)-2 5-Dimethoxy-4-bromoamphetamine | (-)-Ro 363 | (Â±)-(4aS 8R 8aS)-8-amino-2-cyclohexyl-3 4 4a 7 8 8a-hexahydroisoquinolin-1(2H)-one |
| CGP 12177 | Epinephrine ((-)-adrenaline) | 9-OH-risperidone | [3H]dihydroalprenolol | (Â±)-(4aS 8S 8aS)-2-benzyl-N-(4-chloro-3-(trifluoromethyl)phenyl)-1-oxo-1 2 3 4 4a 7 8 8a-octahydroisoquinoline-8-carboxamide |
| Fenoterol (+/-) | Formoterol (+/-) | Brexpiprazole | [3H]L748337 | (Â±)-2 5-Dimethoxy-4-ethylamphetamine |
| Noradrenaline- (-) (Norepinephrine- (-)) | ICI 118, 551 | Bromocriptine | Acebutolol | (Â±)-2 5-Dimethoxy-4-iodoamphetamine |
| Pindolol (+/-) | Indacaterol (R) | Cariprazine | Amibegron | (Â±)-2 5-Dimethoxy-4-methylamphetamine |
| SR 59230A | Alprenolol (+/-) | Clozapine | BRL35135 | (Â±)-3 5-dichloro-N-((4aS 8R 8aS)-2-cyclohexyl-1-oxo-1 2 3 4 4a 7 8 8a-octahydroisoquinolin-8-yl)benzamide |
| Atenolol | Bisoprolol | Fenfluramine (+/-) | Bucindolol | (Â±)-4-bromo-3-(trifluoromethyl)-N-((4aS 8R 8aS)-2-cyclohexyl-1-oxo-1 2 3 4 4a 7 8 8a-octahydroisoquinolin-8-yl)benzamide |
| CGP 20712-A | Propranolol l- | Fluorocarazolol, (R) | Butoxamine | (Â±)-4-bromo-3-(trifluoromethyl)-N-((4aS 8R 8aS)-2-cyclohexyl-1-oxo-1 2 3 4 4a 7 8 8a-octahydroisoquinolin-8-yl)benzamide |
| Metoprolol (+/-) | Salbutamol (+/-) (Albuterol) | Fluorocarazolol, (S) | CHF-6366 | (Â±)-4-chloro-3-(trifluoromethyl)-N-((4aS 8R 8aS)-2-cyclohexyl-1-oxo-1 2 3 4 4a 7 8 8a-octahydroisoquinolin-8-yl)benzamide |
| Propranolol (+/-) | Salmeterol | Fluspiriline | CL316243 | (Â±)-4-chloro-N-(((4aS 8R 8aS)-2-benzyl-1 2 3 4 4a 7 8 8a-octahydroisoquinolin-8-yl)methyl)-3-(trifluoromethyl)aniline |
| Terbutaline |  | Haloperidol | Cyanopindolol | (Â±)-4-Ethylthio-2 5-dimethoxyamphetamine |
| ICI 89, 406 |  | Lisuride | Denopamine | (Â±)-N-(((4aS 8R 8aS)-2-(3 4-dichlorobenzyl)-1 2 3 4 4a 7 8 8a-octahydroisoquinolin-8-yl)methyl)aniline |
|  |  | Lysergic acid (+) | Esmolol | (Â±)-N-((4aS 8R 8aS)-2-benzyl-1-oxo-1 2 3 4 4a 7 8 8a-octahydroisoquinolin-8-yl)-4-chloro-3-(trifluoromethyl)benzamide |
|  |  | MRS5698 | H87/07 | 1-[4-(5-chloro-2-methylphenyl)-1-piperazinyl]-3-(2 5-dimethylphenoxy)-2-propanol dihydrochloride |
|  |  | N-desmethylclozapine | ICI-89406 | 1-1(1-Fluoropropan-2-ylamino)-3-(20cyclohexlpheonxy)propan-2-ol ((2R 2S)-1) |
|  |  | Olanzapine | L 755507 | 1-1(1-Fluoropropan-2-ylamino)-3-(20cyclohexlpheonxy)propan-2-ol ((2S)-1) |
|  |  | ORG-5222 (+/-) (Asenapine (+/-)) | L742791 | 1-methyl-7 8 9 10-tetrahydro-1H-6 10-methanoazepino[4 5-g] quinoxalin-2(6H)-one HCl |
|  |  | Pipamerone | L-748328 | 2-(Dimethylamino)-2-oxoethyl 4â€²-(Piperidin-4-yl)-5-(4-(4-(trifluoromethyl)phenyl)-1H-1 2 3-triazol-1-yl)-[1 1â€²-biphenyl]-3-carboxylate |
|  |  | Quetiapine | L-748337 | 25CN-NBOH |
|  |  | Risperidone | landiolol | 2-Cl-diphenidine |
|  |  | SB204070 | Levosalbutamol | 2-methoxy-diphenidine |
|  |  | Sertindole | NDD-713 | 3-(piperazin-1-yl)-N-(m-tolyl)propanamide |
|  |  | Terguride | NDD-825 | 3-methoxy-diphenidine |
|  |  |  | Olodaterol | 4-Ethylthio-2 5-dimethoxyphenethylamine |
|  |  |  | Orciprenaline | 4-Ethylthio-2 5-dimethoxyphenylbutylamine |
|  |  |  | Practolol | 4-methoxy-diphenidine |
|  |  |  | Prenalterol | 6-APB (6-(2-aminopropyl)benzofuran) |
|  |  |  | Procaterol | Broxaterol |
|  |  |  | Ractopamine | Cis-2a |
|  |  |  | T-0509 | D-578 |
|  |  |  | Tertatolol | Diphenidine |
|  |  |  | Vibegron | DKR-1677 |
|  |  |  | Vilanterol | FEMPT |
|  |  |  | Xamoterol | Fenfluramine (-) |
|  |  |  | Zinterol | Finazine |
|  |  |  | (±)-adrenaline | Isoproterenol (-) |
|  |  |  | 7-methylcyanopindolol | Isoproterenol (+) (Isoproterenol-l) |
|  |  |  | Betaxolol | KUR-1246 |
|  |  |  | BI-167107 | mCPP |
|  |  |  | Cicloprolol | MRP3160 |
|  |  |  | Clenbuterol | MRP3172 |
|  |  |  | labetalol | MRP3215 |
|  |  |  | LK 204-545 | MRS5676 |
|  |  |  | NIHP (1-[4-(3-hydroxypropyl)phenoxy]-3-(propan-2-ylamino)propan-2-ol)HP | MRS5678 |
|  |  |  | NIP (N-isopropyl-phenoxypropanolamine) | MRS5697 |
|  |  |  | Noradrenaline- (+/-) (Norepinephrine- (+/-)) | MRS5755 |
|  |  |  | Propafenone | N-(3-chlorophenyl)-3-[4-(3-phenyl-2-propen-1-yl)-1-piperazinyl]propanamide |
|  |  |  | Sotalol | N-(3-methylphenyl)-3-[4-(3-phenyl-2-propen-1-yl)-1-piperazinyl]propanamide hydrochloride |
|  |  |  | Timolol | Naftopidil |
|  |  |  | [^125^I]ICYP | S S-2c |
|  |  |  | Abediterol | TMQ (+/-)-8-Fluoro (8-fluorotrimetoquinol) |
|  |  |  | Bunolol |  |
|  |  |  | Bupranolol |  |
|  |  |  | Carazolol |  |
|  |  |  | Cimaterol |  |
|  |  |  | Isoprenaline |  |
|  |  |  | Mirabegron |  |
|  |  |  | Nadolol |  |
|  |  |  | Nebivolol |  |
|  |  |  | Solabegron |  |
